# Substituent Effect in the Cation Radicals of Monosubstituted Benzenes

**DOI:** 10.3390/ijms22136936

**Published:** 2021-06-28

**Authors:** Jan Cz. Dobrowolski, Wojciech M. Dudek, Grażyna Karpińska, Anna Baraniak

**Affiliations:** 1 National Medicines Institute, 00-725 Warsaw, Poland; g.karpinska@nil.gov.pl (G.K.); a.baraniak@nil.gov.pl (A.B.); 2 Institute of Nuclear Chemistry and Technology, 03-195 Warsaw, Poland; w.dudek@ichtj.waw.pl

**Keywords:** benzene derivatives, cation radicals, substituent effect, DFT, pEDA, sEDA, ionization potential, aromaticity, spin distribution, charge distribution

## Abstract

In 30 monosubstituted benzene cation radicals, studied at the ωB97XD/aug-cc-pVTZ level, the phenyl rings usually adopt a compressed form, but a differently compressed form—equivalent to an elongated one—may coexist. The computational and literature ionization potentials are well correlated. The geometrical and magnetic aromaticity, estimated using HOMA and NICS indices, show the systems to be structurally aromatic but magnetically antiaromatic or only weakly aromatic. The partial charge is split between the substituent and ring and varies the most at C(ipso). In the ring, the spin is 70%, concentrated equally at the C(ipso) and C(p) atoms. The sEDA(*D*) and pEDA(*D*) descriptors of the substituent effect in cation radicals, respectively, were determined. In cation radicals, the substituent effect on the σ-electron system is like that in the ground state. The effect on the π-electron systems is long-range, and its propagation in the radical quinone-like ring is unlike that in the neutral molecules. The pEDA(*D*) descriptor correlates well with the partial spin at C(ipso) and C(p) and weakly with the HOMA(*D*) index. The correlation of the spin at the ring π-electron system and the pEDA(*D*) descriptor shows that the electron charge supplied to the ring π-electron system and the spin flow oppositely.

## 1. Introduction

Under normal conditions, most molecules have an even number of electrons, all paired. The manifolds of orbitals with identical energy are, at the single reference level, either entirely filled or empty. They are the closed-shell molecules. On the other hand, those molecules which have either an odd number of electrons or have some orbitals singly occupied are the open-shell molecules, i.e., radicals, di- or biradicals, etc. It is more difficult to indicate the chemical disciplines in which the role of radicals is marginal than those for which it is considerable, e.g., [1,2,3,4,5,6,7,8,9,10]. Indeed, radicals appear in many organic reactions [11,12], inorganic coordination complexes in which metal ions have unpaired electrons [13,14,15], and modern industrial processes [16], i.e., radical polymerization [17]. Moreover, radicals play the essential role in a whole host of various disease states and their perpetuation [18,19], including cancer and DNA damage [20,21,22], skin aging [23], and cardiovascular [24], neurodegenerative [25,26], and immunological diseases [27], as well as atherosclerosis, diabetes, dyslipidemia [28], etc. However, radicals are also studied to improve the diagnostic and therapeutic capabilities of various medicines, e.g., [29,30,31,32,33,34,35,36,37].

Cation radicals need an energy supply to be formed [38]. For aromatic hydrocarbons it is ca. 200 kcal/mol [39]. However, they can be formed as ordinary as after dissolving in sulfuric acid, and besides sulfonation the one-electron oxidation occurs, too. Cation radicals can also be generated using organic (e.g., persulfate, iodosylbenzene) and inorganic (e.g., H_2_O_2_, ClO_2_, SbCl_5_, AlCl_3_, I_2_, XeF_2_) reagents as well as metal ions (e.g., Tl(III), Mn(III), Co(III), Ce(IV), Ag(I), Ag(II), Pd(II)) [38]. A convenient way to prepare cation radicals is anodic oxidation in inert solvents, which in aromatic hydrocarbons is the one-electron detachment from the π-electron system. They are not stable, and in the subsequent anodic reaction, the second electron or a proton can be lost to form a dication or a neutral radical. Cation radicals are also formed as a response to radiolysis [40,41,42,43]: photolysis or ion or particle-beam irradiation; external alpha, beta, and gamma radiation; or radiation by the embedded radionuclides, etc. Different types of radiation produce direct proteins, carbohydrates, DNA and RNA damage, or indirectly destroy the biomolecules by generating water radical ions [43,44,45,46,47]. Similar to monosubstituted benzenes, which are archetypal for monosubstituted aromatics, monosubstituted benzene monocation radicals constitute a reference for cation radical aromatics including PAHs, for which there is evidence for the involvement of one-electron oxidation in PAH carcinogenesis [48,49,50,51,52]. This is why in this study we focus on monosubstituted benzene cation radicals.

The substitution of a chemical system by a functional group is probably the most fundamental modification of a molecule in the search for the more desirable properties of a compound in the target situation. The substitution is also a fundamental chemical operation allowing for the understanding of the compound properties. The effect of substitution in closed-shell systems has been studied since the first half of the 20th century and is well recognized [53,54,55,56,57,58,59,60,61,62,63,64,65,66,67,68,69]. It can be quantitatively evaluated by an array of parameters [60,61,62]. However, for the excited states, the number of specially designed substitution effect descriptors is small: the σ^ex^_CC_ type of descriptors were proposed by the Cao group [63,64,65,66,67,68,69,70,71], the cSAR(ex) one was defined by Sadlej-Sosnowska and Kijak [72], and the sEDA(*S*_1_) and pEDA(*S*_1_) as well as sEDA(*T*_1_) and pEDA(*T*_1_) parameters were constructed by us for the first singlet and first triplet states, respectively [73,74].

By contrast, quite a number of substituent parameters for radical reactions were elaborated [75,76,77]. However, in the Hansh and Gao critical review on QSAR analyses of radical reactions of benzene derivatives [76], the attention was focused around the classical σ [53,78] and the modified (σ^+^ and σ^−^) [79,80] Hammett constants. The σ^+^ and σ^−^ constants were defined by non-radical reactions with positive or negative charges at the reaction center. The fair performance of the σ, σ^+^, and σ^−^ in analyses of radical reactions was assigned to the significant contribution of the cationic or anionic character of the radical reactions. Hansh and Gao also considered descriptors designed specifically for radicals: the Azumi and Yamamoto (*E*_R_) resonance constant [81] and the Dust and Arnold σ^•^ [82,83], as well as Cerary et al.’s [84,85] and Jiang and Ji’s [86] versions of the σ^•^ descriptor. Most of the considered descriptors were derived from the kinetic studies of different organic (radical) reactions, while the Dust and Arnold σ^•^ scale was based upon the electron spin resonance hyperfine coupling constants in the meta and para positions of benzylic radicals on reflected spin delocalization [82,83]. However, the modified σ^•^_C_ scale of Cerary et al. was constructed based on the thermal rearrangement rate of substituted methylenecyclopropanes to the isopropylidenecyclopropanes and correlation with the original σ^•^ scale [85]. On the other hand, the Jang and Ji σ^•^_JJ_ scale was based on ^19^F-NMR data for the rate constants of the substituted trifluorostyrene cycloaddition reactions [86]. Although the *E*_R_ and σ^•^ parameters were specially constructed for radicals, Hansch and Gao stated that they could not generalize the performance of these parameters since there was “*a limited number of substituents for which they have been determined*” [76]. In 2000, Héberger, Lopata, and Jászberényi argued that σ described polar effects in radical addition reactions, and σ^•^ of Cerary did this to a lesser extent, while σ^•^ of Dust and Arnold described the enthalpy effects [87]. For a small review of further development, see the Supplementary Information file.

The Hammett-like substituent effect constants and the most important scales defined for radicals are based on disubstituted benzene ring molecules, either in para or in meta position. We have constructed series of the sEDA and pEDA kind substituent effect descriptors based on monosubstituted benzenes [62,73,74]. Since the first sEDA and pEDA descriptors were defined to evaluate the classical substituent effect [62], we have constructed several analogous descriptors to evaluate the substituent effect in the first excited singlet and first excited triplet states [73,74] substitution by a group bonded by a double bond [88] and the heteroatom incorporation effect in single five- and six-membered rings and at the ring-junction position of cumulated rings [89,90]. The definition of the descriptors is based on comparison between the amount of the σ-valence electron charge (sEDA) or π-valence electron charge (pEDA) in the monosubstituted benzene derivative and the analogous amounts in the unsubstituted benzene. Estimation of the σ and π valence orbital populations is evaluated by the NBO method [91,92,93], as implemented in the Gaussian program. The descriptor values express the amount charge shifted between the substituent and the benzene ring within the σ-valence electron system (sEDA) and π-valence electron system (pEDA) [62]. In all studied systems, either in the ground or excited states, single or double bond substituted, or with an inserted group, the sEDA type of descriptors define substituent electronegativity for a molecule in a given state. The sEDA descriptors strongly and linearly correlate with the classical Pauling or Milliken electronegativities, but reflect the effect on the σ-electron structure more smoothly. This is because the sEDA values vary with the substituent structure, while the Pauling or Milliken ones are determined by the kind of atom through which the substituent is attached/incorporated into the ring. The pEDA descriptors express influence on the delocalized π-electron structure and correlate with the resonance effect descriptors.

The aim of this paper is four-fold. First, to construct sEDA(*D*) and pEDA(*D*) substituent effect descriptors evaluating the substituent effect on the σ- and π-electron structure of cation radicals. Second, to evaluate cation radicals’ aromaticity in more detail. Third, to look into the influence of the substituent on spin contribution in the radicals. Finally, to check whether the restricted open-shell (RO) and unrestricted (U) approximations combined with the solid DFT level, such as (ωB97XD/aug-cc-pVTZ), will provide concordant results about the properties of monosubstituted benzene cation radicals. However, the necessary computational steps have molded the organization of this paper, in which optimized structures are discussed first, then energetic and ionization potentials are described, next aromaticity is analyzed, and finally partial charge and spin distributions are examined. The comparison of RO- and U-DFT results is addressed in each subsection. Such a combined description has not been presented before.

## 2. Results and Discussion

### 2.1. Geometry 

High reactivity and a short lifetime make the structural characterization of cation radicals very challenging. The structures of some cation radicals can be determined by the X-ray diffraction of their solid state salts with weakly coordinating anions [94,95,96]. Yet, the geometries of most radicals are indirectly obtained via fast, time-resolved, spectroscopic techniques (the first being ESR, UV-Vis, IR) accompanied by computations. Nowadays, transient absorption spectroscopy with high-order harmonic generation (HHG) sources [97,98,99] is an especially successful technique in the determination of the cation radical structure and dynamics. It is free from the uncertainty constraint, so both the timing and the spectral resolution are independent and registered separately. Therefore, it couples the possibility of attosecond measurements with the atomic specificity of X-ray spectroscopy.

**Scheme 1:**
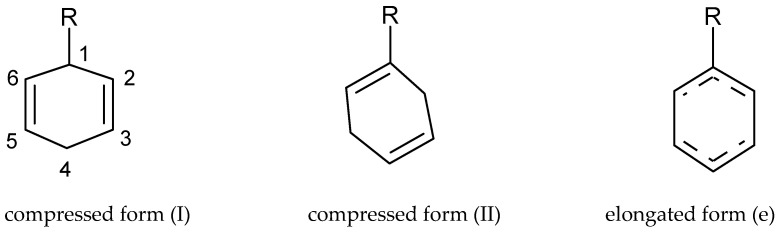
Three forms of substituted benzene cation radicals calculated using the U- and RO-DFT approximations and the ωB97XD/aug-cc-pVTZ level.

Quite recently, the structure of the benzene cation radical has been elucidated by Table-Top X-ray spectroscopy accompanied by high-level equation-of-motion coupled-cluster calculations [100,101]. It appears that in the benzene cation radical, there is a splitting of the two degenerate π* orbitals manifested by a new peak coming from an excitation to the partially occupied π-subshell. The splitting has been interpreted as being due to the radical cation distortion and serious dominant spin coupling of the electrons unpaired at partially and fully vacant π* orbitals. As a result, the compressed (^2^*B*_2g_) and elongated (^2^*B*_3g_) benzene radical cation structures (Scheme 1) have been assigned and predicted to differ in energy by only ca. 15 cm^−1^, i.e., by the value which is below the zero-point energy, and presumably, also below the high-level computation accuracy.

Here, irrespective of whether the U- or RO-approximation has been used, the ωB97XD/aug-cc-pVTZ calculations predict the compressed benzene cation radical ((I), Scheme 1) to be stable and the elongated one ((e), Scheme 1) to be a transition state with one imaginary frequency and to have higher energy than that of the compressed structure by 112 cm^−1^ (U-DFT) or 85 cm^−1^ (RO-DFT). The compressed form has a quinoid-like ring with a decreased C1–C4 distance and C2–C3 and C5–C6 bond lengths shorter than the other ring’s CC bonds. In the elongated form, the C1–C4 distance and the C2–C3 and C5–C6 bond distances are longer than the other CC bonds in the ring. The compressed structure is also predicted for almost all monosubstituted benzene cation radicals.

However, for the CHO and COOH substituted benzene radicals calculated with the RO-DFT method, beside the form (I) compressed along the C1C4 axis, a form (II) compressed along the C2C5 axis is found. However, for CHO it is less stable than (I) by ca. 0.5–1.3 kcal/mol, but for COOH, the form (II) is more stable than (I) by as much as 15.0 kcal/mol (Table S1a,b). For the acetophenone cation radical, two forms (I) differing in energy by 1.4 kcal/mol have been found too, but only with the U-DFT approximation. In the more stable form, the C(ipso)–C(OCH_3_) bond is shorter and thus has a more significant double bond contribution. Moreover, in the case of the NO_2_ substituent, the presence of two stable forms (I) is found. One has the NO_2_ group slightly skewed with respect to the benzene ring and the C–N distance corresponding to the single bond. However, in the other, less stable one, one sees the NO_2_ group coplanar with the benzene ring and the C–N distance corresponding to the double bond. For the BF_2_ substituent, our calculations provide only form (II). In the end, the Li and Na benzene cation radicals and the metal cations dissociate and drift over the benzene plane.

Now, notice that the form (II) is discovered in systems which are asymmetric with respect to the perpendicular plane containing the C1C4 axis, i.e., the CHO, COOH, and CF_3_ derivatives (where in CF_3_ one CF bond is coplanar with the ring). Yet, in form (II), a rotation about the single C–R bond inverts the single/double bond pattern into the reciprocal one (as in the resonance structure). The barrier for such a rotation is ca. 350 cm^−1^ (CF_3_) or 650 cm^−1^ (CHO, COOH), which is above the RT factor in 300 K equal to 208 cm^−1^. However, it seems that the fluctuation of charge with the change in the bond lengths in the asymmetric C6=C1–C2 stretching vibrations, contributing to ring deformation modes absorbing at ca. 1400 cm^−1^, exceeds the barrier and easily inverts the single/double bond pattern in the ring. Thus, the true bond length motif can be considered as the average over the pairs of reciprocal bonds: C1C2 and C1C6; C2C3 and C6C5; and C3C4 and C5C4. As a result, the bond scheme becomes similar to that in the elongated form: 1.4012, 1.4055, 1.3975 Å (RO-DFT) for CHO; 1.3932, 1.421, 1.3910 Å (U-DFT) and 1.3928, 1.4200, 1.3905 Å (RO-DFT) for COOH; and 1.3917, 1.4207, 1.3913 Å (U-DFT) and 1.3914, 1.4194, 1.3910 Å (RO-DFT) for CF_3_, where the first, second, and third value correspond to the mean of the C1C2 and C1C6; C2C3 and C6C5; and C3C4 and C5C4 bonds. 

Summing up the above, most of the substituted benzene cation radicals were found to have the compressed, quinoid-like, form (I) of the ring. For a few derivatives, a second compressed quinoid-like form (II), or an elongated one which could coexist with the form (I), was also predicted. We argue that in fact there are two forms (II), which are two resonance structures that cannot be automatically averaged by computations. Thus, the prediction of the presence of the form (II) is equivalent to the prediction of the existence of the elongated form. 

Still, can different forms of the same substituted benzene cation radical coexist? To rationally argue for a real coexistence of different forms, the heights of the barriers between them must be higher than their zero-point energies. Otherwise, the forms only contribute in a deformation of the potential energy well. Yet, finding TSs between different radical forms goes beyond the aim of this study and should be the subject of calculations performed at a much higher theoretical level. 

However, if the band splitting observed for the benzene monocation radical [100,101] has been correctly interpreted as a manifestation of a coexistence of two radical forms, then the resonance isomers have been observed for the first time. The famous Pauling’s Resonance Theory [102] refers to the electronic structure of a π-electron molecule, which is a superposition of several virtual, inseparable, structures whose wave functions contribute to its entire wave function. As far as we know, no such isomers, also referred to as mesomers [103], have ever been observed in the ground state molecules. The resonance isomers can be defined as detectable stereoisomers [104] in the same electronic state, but with distinct charge redistributions. The requirement of the same electronic state is necessary because otherwise the same molecule in various electronic states would fulfill the definition [105]. Taking all the above into account, the present study may indicate that if the resonance isomers of the monosubstituted benzene cation radicals really exist, then they could possibly be found more easily in the CHO and COOH benzene derivatives. Still, it is also possible that at a higher level of theory the presence of the resonance isomers of benzene cation radicals would be excluded. At this moment, it is reasonable to treat the prediction of different geometrical forms of radical cations with a certain reservation.

### 2.2. Energetics 

**Figure 1:**
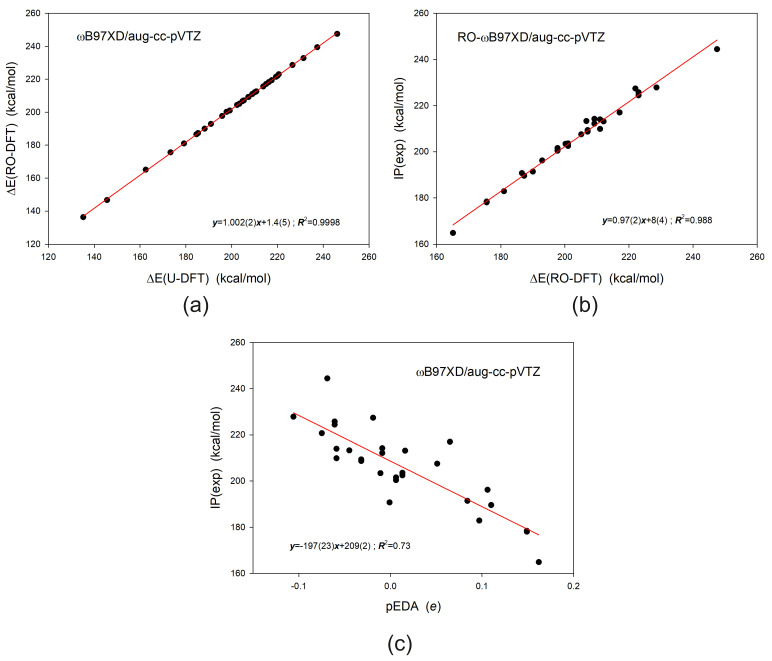
(**a**) The total energy differences ΔE (kcal/mol) of the monocation radicals of monosubstituted benzenes calculated using the U- and RO-DFT approximations referring to the ground singlet state total energies calculated using the R-DFT method. (**b**) Linear correlations between experimental ionization energies and the relative values of total energies (ΔE, kcal/mol) of monosubstituted benzene monocation radicals calculated using RO-DFT approximations and the ωB97XD/aug-cc-pVTZ level, referring to the energies of the molecules in the ground singlet state calculated at the same level. (**c**) Weak linear correlations between experimental ionization energy and the pEDA substituent descriptor reflecting substituent influence on the benzene π-electron system in the ground state. All IP(exp) values (Table 1) were considered in plots (**b**,**c**).

The energies were calculated using the unrestricted (U) and restricted open-shell (RO) DFT approximations, the ωB97XD functional and the aug-cc-pVTZ basis set (Table S1a–f). The U- and RO-DFT calculated energies are largely congruent with respect to both: (i) the optimized total energies referring to the ones of the ground states (Figure 1a), and (ii) the single point energies obtained for the fixed ground state geometries (Figure S1a). The latter corresponds to the vertical processes with an immediate electron loss and conservation of the geometry of the unperturbed ground singlet state. Next, the charge and spin redistributions drive the monocation radical geometry towards the relaxed structure. The vertical ionization energy is about 200 kcal/mol, while the relaxation energy is about 7. kcal/mol, except for some forms for which it approaches 25 kcal/mol (Figure S1c–f, Table S1f).

The cation radical and the ground state total energy differences are in agreement with the experimental IP values [106,107,108,109,110,111,112,113,114,115,116,117,118,119,120,121,122,123,124,125,126,127,128] (Table 1, Figure 1b and Figure S1b). The rows in Table 1 contain three types of data for which: (i) the experimental values were determined and only one molecular form was found in the calculations, (ii) the computations predict more than one form, and (iii) the experimental values were not found. Types (i) and (iii) require no comments. Agreement between computational values and the experimental IP data offers a chance to recognize the experimental geometry (Table 1).

**Table 1:** Comparison of relative values of the total energies (ΔE, kcal/mol) of monosubstituted benzene monocation radicals.

U-ωB97XD		RO-ωB97XD		Exp.	
Substituent	ΔE	Substituent	**ΔE**	**IP**	Ref.
BF_2_ (II)	216.2	BF_2_ (II)	218.2		
BH_2_ perp	210.8	BH_2_ perp	212.6		
B(OH)_2_	202.4	B(OH)_2_	204.4		
Br	203.4	Br	205.1	207.54	[106,107]
CCH	197.8	CCH	200.2	203.39	[108]
CF_3_ (II)	220.0	CF_3_ (II)	222.0	227.38	[109]
CFO	246.1	CFO	247.5	244.44	[110]
CH_3_	199.2	CH_3_	201.0	203.57, 202.47	[111,112]
CHO (I) LOC	218.9	CHO (II)	219.4	220.69	[113]
CHO (I) MIN	217.5				
Cl	205.3	Cl	207.1	209.39,208.70	[106,107]
CN	220.6	CN	223.0	224.41; 225.76	[112,113]
COCH_3_ LOC	210.6	COCH_3_	211.0	214.00;209.90	[114,115]
COCH_3_ MIN	209.0				
CONH_2_	204.8	CONH_2_	206.7	213.31	[110]
COOH (II) MIN	215.1	COOH (II) MIN	217.1	217.00	[112]
COOH (I) LOC	231.4	COOH (I) LOC	232.8		
F	207.4	F	209.2	212.16; 214.23	[106,107]
H	210.1	H	212.1	213.17	[117]
Li	145.6	Li	146.7		
MeSO_2_	219.4	MeSO_2_	221.5		
Na	135.2	Na	136.3		
NC	213.8	NC	215.6		
NH_2_	173.3	NH_2_	175.6	178.40;178.04	[118,119]
Nme_2_	162.5	Nme_2_	165.1	164.88	[116]
NO_2_ (I)	237.4	NO_2_ (I)	239.4	227.84	[112]
NO_2_ (I) skew MIN	226.6	NO_2_ (I) skew MIN	228.6		
OH	191.0	OH	192.9	196.20;183.09	[120,121]
Ome	185.3	Ome	187.3	189.60;189.56;193.48	[112,122,123]
Ph	184.6	Ph	186.6	190.71;179.41	[124,125]
SH	188.2	SH	190.0	191.40	[126]
SiH_3_	207.3	SiH_3_	209.2		
Sme	179.2	Sme	181.0	182.87	[116]
tBu	195.9	tBu	197.7	200.40;201.55	[127,128]

Calculated using the U- and RO-DFT approximations and the ωB97XD/aug-cc-pVTZ level referring to the energies of the molecules in the ground singlet state calculated at the same level with the experimental values of ionization energies (IP, kcal/mol, rounded to 2 decimal places). Most of the systems have the compressed geometry of the ring. When more than one form is predicted or a form adopts a different geometry, the abbreviations are introduced: (I) and (II) denote two different compressed forms (Scheme 1), perp or skew denote perpendicular or skewed positions of the substituent with respect to the ring, MIN and LOC stand for the most stable and local minimum forms, and perp and skew denote perpendicular and skewed positions of the substituents. Let us additionally remark that comparison with the Zero-Point Energy (ZPE) corrected values would be methodologically more rigorous. However, the ZPEs correlate very well with the total energy differences. Additionally, for a careful analysis of the IP values, inclusion of both the anharmonic corrections and analysis of orbital excitations would be desirable. Yet, a rigorous approach would go far beyond this study, and therefore, the IP analysis presented here is more qualitative than quantitative.

Indeed, the comparison of the computational and experimental data suggests which of the forms of the COOH and NO_2_ substituent benzene cation radicals exist. The COOH (II) form (Scheme 1) is predicted to be much more stable than the COOH (I), although it has an IP similar to the experimental one (Table 1 and Table S1a,b). Additionally, the NO_2_ substituted benzene cation radical with the nitro group skewed against the benzene plane is more likely to correspond to the experimental form than that with the co-planar NO_2_ group. Moreover, when the U- and RO-DFT calculations concordantly suggest a certain IP value and several experimental Ips are determined in different studies, as, e.g., for biphenyl (Ph) [126,127], the calculations indicate which of the experimental values can be the most accurate.

However, for the CHO substituent, a small energy difference between the calculated forms makes such an indication impossible. At the end of this section, let us notice the weak linear correlations between the experimental ionization potentials, or the calculated energy differences, and the ground state pEDA substituent descriptor [62] reflecting the substituent influence on the benzene π-electron system (Figure 1c and Figure S1g).

### 2.3. Aromaticity

#### 2.3.1. Geometrical Aromaticity

In the monosubstituted benzenes in the ground singlet *S*_0_ state, the phenyl ring is planar. The π-valence electron structure and the ring geometry are only weakly perturbed by the substituents. This indicates a resistance of the π-electron structure to the mono- and homo-disubstitution. Indeed, the HOMA (Harmonic Oscillator Model of Aromaticity) geometrical aromaticity index [129,130] of monosubstituted and meta and para homo-disubstituted benzenes varies little with the substituent and is only slightly lower than 1 [131,132,133,134]. Nevertheless, the effect of six identical substituents can be significant [135]. In the monosubstituted benzenes in the first excited singlet *S*_1_ state, the ring is at most slightly distorted [73]. Yet, in the first excited triplet *T*_1_ state, it is significantly deformed [74]. Thus, the monosubstituted benzenes are geometrically aromatic in the ground state (HOMA ≈ 1), less aromatic in the *S*_1_ state (HOMA ≈ 0.8), and either nonaromatic or slightly antiaromatic in the *T*_1_ state (HOMA ≈ 0.0 ± 0.4) [74]. 

**Figure 2:**
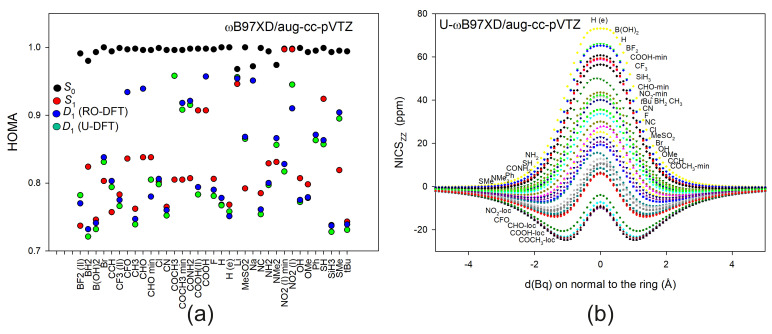
(**a**) A variation of the HOMA aromaticity indices of the monosubstituted benzenes in the S0 ground (black), S1 first excited singlet (red) [74], and the cation radical D1 doublet states (blue and green) calculated using the aug-cc-pVTZ basis set and the ωB97XD, TD-ωB97XD, and U- and RO-ωB97XD method, respectively. (**b**) The NICSZZ scans in monosubstituted benzene cation radicals plotted against sample point distance d(Bq) from the normal to the benzene ring passing through the ring center, which is placed in the origin. The molecule is located in the XY plane. The calculations were performed at the U-ωB97XD/aug-cc-pVTZ level.

The HOMA index of phenyl rings in the studied monocation radicals exceeds 0.7 (Figure 2), which indicates that these rings are aromatic. Although the radicals are visibly less aromatic than the neutral parent molecules, they are about as aromatic as the monosubstituted benzenes in the first excited singlet states [73] (Figure 2) and much more aromatic than in the first excited triplet states [74]. Moreover, the HOMA of the studied radicals is larger than that of any n-acene ring for *n* > 3 [136,137], as well as the non-terminal rings in cata- and peri-polycondensed aromatic hydrocarbons [138]. This is in line with the surprising Rosokha and Kochi finding that the aromaticity of some arene cation and anion radicals can be higher than that of the neutral parent species [139].

The decrease in aromaticity in comparison to the parent neutral compounds occurs because the cation radical rings adopt a quinoidal-like form with the bond length alternation pattern similar to that in non-aromatic systems. However, some cation radicals have HOMA > 0.9. In the case of Li and Na, the higher aromaticity is due to the dissociation of the metal ion and its drift over the ring plane. This releases the quinoidal bond arrangement and promotes charge delocalization and ring aromatization. The most stable COOH derivative has (II) form, which is also quinoidal (HOMA ≈ 0.78, Table S2a). Still, in the less stable quinoidal form (I), the C(ipso)–C(OOH) bond is much shorter than in the form (II), the C1–C2 and C1–C6 bonds are single, and the other four bonds have similar lengths despite the fact that C2–C3 and C5–C6 are a bit shorter than those incident to the C4 atom. As a result, the HOMA index of this form exceeds 0.9 (Table S2a). Analogous situations hold true for the less stable CHO derivative (Table S2a), the only form found for CFO, and the less stable COCH_3_ derivative calculated with the U-DFT method. Finally, the less stable NO_2_ form also exhibits HOMA > 0.9 for the same reason, while the most stable one has HOMA close to 0.8 (Figure 2a). Therefore, for the substituted benzene radical cations, geometrical aromaticity is not the major factor determining molecular stability. To better inspect the causes of the specific ring deformation, let us examine charge and spin distribution in benzene cation radicals.

#### 2.3.2. Magnetic Aromaticity

Except for HOMA, the most popular geometrical aromaticity index is the NICS (Nucleus-Independent Chemical Shift) magnetic aromaticity index, defined as a reverse chemical shift value calculated at the sampling point either in the middle of the ring or 1 Å above it [140]. NICS<<0 indicates that the ring is aromatic, while NICS>>0 indicates that it is antiaromatic (yet, the border between aromatic/antiaromatic and non-aromatic species is fuzzy [141]). In contrast to HOMA, which is a relative index reaching its maximum = 1.0 for benzene being the standard, NICS is not a relative index and has neither an upper nor a lower limit. There are several versions of the NICS(1) index enabling the filtering of the valence σ-orbitals’ contribution to the NICS index, whose aim is to determine only the effects originating from the π-electron system [142]. A more exact profile can be sketched through the NICS function of the sampling point distance from the ring center at the normal to the ring [143,144]. It is worth adding that besides HOMA and NICS there is a variety of aromaticity descriptors which reveal the other aspects of this complex property. Thus, a complementary perspective can be obtained by determining, e.g., [145], measures of the electron-delocalization such as the aromatic fluctuation index (FLU) [146,147] and para-delocalization index (PDI) [148,149]. Still, in this study we restrict aromaticity analysis to the geometrical and magnetic HOMA and NICS indices. The NICS_ZZ_ index is determined from the ZZ diagonal element of the shielding tensor in probing points for the molecule oriented in the XY plane and the origin in the ring center.

**Figure 3:**
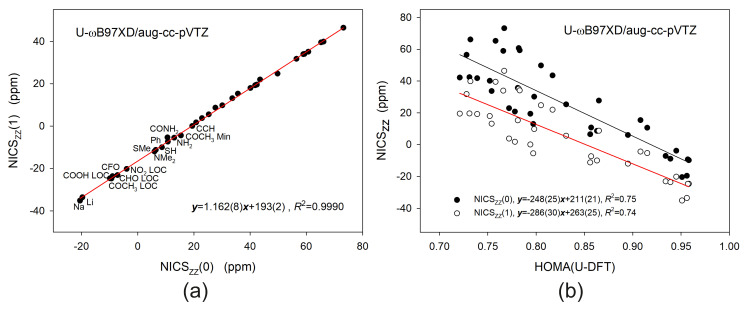
(**a**) Strong linear correlation between the NICS_ZZ_(1) and NICS_ZZ_(0) indices calculated for the monosubstituted benzene cation radicals. The forms with NICS_ZZ_(1) < 0 are explicitly signed. (**b**) Weak linear correlations between HOMA and NICS_ZZ_(1) and NICS_ZZ_(0) indices calculated for the monosubstituted benzene cation radicals. The calculations were performed at the U-ωB97XD/ aug-cc-pVTZ level.

Similarly to HOMA, for monosubstituted benzenes in the ground state, the NICS indices vary only a little with the substituent (Figure S2). Indeed, for the studied set of substituents, the NICS(1) values range within 5 ppm, i.e., from ca. −26 to ca. 31 ppm (Figure S2, Table S2b). For the ground state, the NICS_ZZ_(0) and NICS_ZZ_(1) are only weakly linearly correlated (Figure S2b). For the cation radicals, in line with HOMA, the NICS indices change significantly: the NICS_ZZ_(0) values range within 100 ppm (Figure 2b, Table S2b, Figure S2c). The NICS_ZZ_(0) and NICS_ZZ_(1) are strongly correlated (Figure 3a) and weakly correlated with HOMA (Figure 3b). However, in contrast to HOMA, the positive NICS_ZZ_(0) values indicate that most of the studied cation radicals are magnetically antiaromatic (Figure 3, Table S2b).

Even if for some cation radicals the NICS_ZZ_(0) is lower than 0 (Table S2b, Figure 3a), these systems (excluding CFO and Li plus Na) are not global minima, and in the overall analysis, can be skipped. Yet, the analysis of the NICS_ZZ_(1) values gives less unequivocal conclusions. The NICS_ZZ_(1) values corresponding to global minima range from ca. −10 to ca. 45 ppm, indicating that the systems are more aromatic than would be suggested by the NICS_ZZ_(0) values (Figure 3, Table S2). Except for the mentioned local minima, CFO, Li and Na, NICS_ZZ_(0) < 0 for SMe, NMe_2_, Ph, SH, CONH_2_, NH_2_, and the most stable form of the COCH_3_ substituted benzene.

Notice that these substituents have a reservoir of electrons in the lone electron pairs which can be shifted to the ring. However, the NICS_ZZ_(1) point is usually placed at the NICS_ZZ_(r) function shoulder (Figure 2b), and r = d(Bq) is the Bq sample point distance to the benzene ring on the normal passing through the ring center placed in the origin of the coordinate system. Moreover, because the shape of the NICS_ZZ_(r) function varies significantly with the substituent (Figure 2b), the family of the NICS_ZZ_(r) functions is apparently governed by several parameters. Therefore, the question as to how well the NICS_ZZ_(1) value reproduces the magnetic aromaticity properties of the system is difficult to precisely address. Nevertheless, assuming that HOMA correctly predicts that the aromaticity of the studied cation radicals is not significantly lower than that of the ground state derivatives, then the NICS_ZZ_(1) changes disagree less with this conclusion than those of NICS_ZZ_(0) (Figure 3b).

In conclusion to the Aromaticity section, we can state that monosubstituted benzene cation radicals are interesting compounds which, although are geometrically aromatic, in terms of the HOMA index, are simultaneously—in the majority—magnetically antiaromatic, in terms of the NICS group of indices. This is yet another example showing that aromaticity is a complex and multidimensional concept [150]. The inhomogeneity of the charge and spin distribution in the ring can shed some light on the cause of magnetic antiaromaticity in the majority of the studied cation radicals.

### 2.4. Charge and Spin Distribution

**Figure 4:**
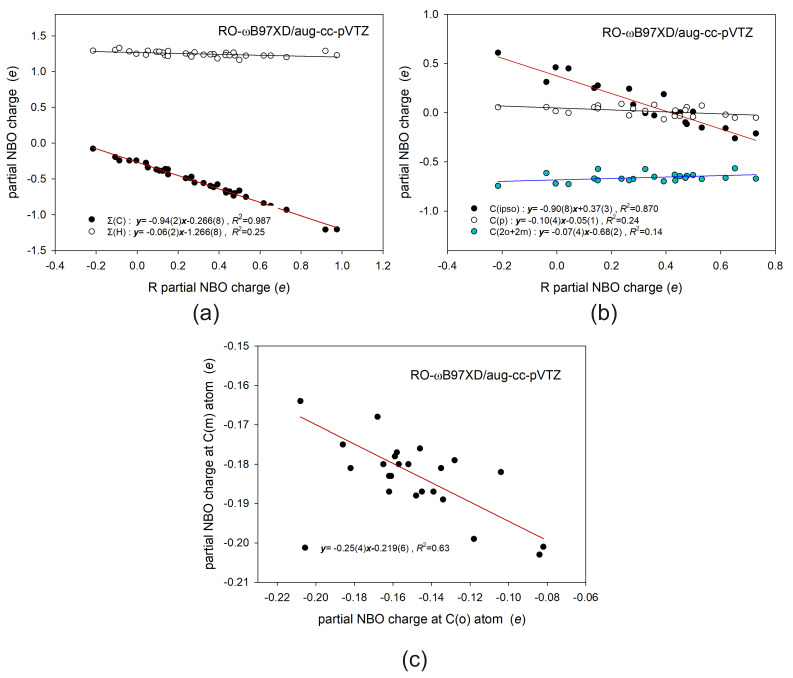
(**a**) Linear changes in the sum of the partial NBO charges at the ring C atoms, Σ(C), and lack of similar changes in the sum of charges at the ring H atoms, Σ(H), in the monosubstituted benzene cation radicals with the change in the charge at all R substituent atoms. (**b**) Linear changes in the partial NBO charge at the C(ipso) atom and lack of similar changes at the C(p), or sum of the ortho and meta C atoms, with the change in the charge at the R substituent. (**c**) Weak linear correlation between the partial charge at C(m) and C(o) atoms. All atypical systems (forms (II) and (e)) were omitted. Calculations were performed using the RO-DFT method.

In benzene monocation radicals, the sum of the NBO partial charges of the five ring H atoms is nearly constant (ca. 1.25 *e*, Figure 4a, Table S3a–d). The partial NBO charge spread over the R substituent atoms and the ring C atoms (ΣC) varies within ca. 1.15 *e*, from about −0.2 to 1.2 for R and −0.0 to −1.2 for the six ring C atoms (Table S3a–d). The charges at R and ΣC are strongly linearly correlated (*R*^2^ = 0.987, Figure 4a). In the ring, the charge at the C(ipso) atom varies the most with the change of the R substituent, and they are linearly correlated (*R*^2^ = 0.87, Figure 4b).

**Figure 5:**
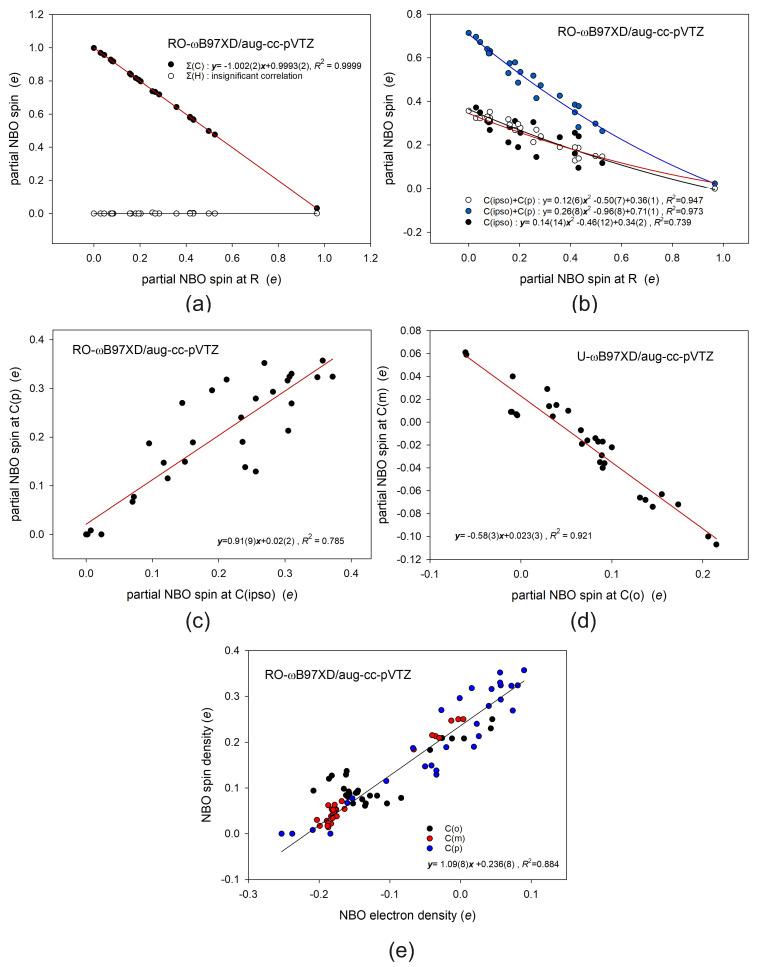
(**a**) Linear changes in the sum of the partial NBO spins at the ring C atoms, Σ(C), and lack of similar changes at the sum of ring H atoms, Σ(H), in the monosubstituted benzene cation radicals with the partial spin at all R substituent atoms. (**b**) Slightly nonlinear correlations of the partial NBO spin at the sum of C(ipso) and C(p) atoms with the partial spin at R and similar, but weaker, correlations for separate C(ipso) and C(p) atoms. (**c**) A fair linear correlation between partial spin located at C(ipso) and C(p) atoms calculated using RO-DFT method. (**d**) A significant linear correlation between partial spin located at C(o) and C(m) atoms calculated using the U-DFT method. All atypical systems (forms (II) and (e)) were omitted in correlations. (**e**) The correlation between NBO electron density and NBO spin density at the ring C atoms.

On the other hand, changes in the C(p) or the sum of the ortho and meta C atom charges with R are too weak to be statistically significant (Figure 4b). Interestingly, the partial charges at the C(o) and C(m) atoms are weakly linearly correlated (*R*^2^ = 0.63, Figure 4c). The two U- and RO-DFT approximations reveal the same relationships between the partial charges in radical cations (Figure S3). Despite ca. 1.2 *e* being located at the ring H atoms, no partial spin is predicted to reside at these very atoms (Figure 5a, Table S4a–f). Thus, the spin at all substituent atoms is perfectly linearly correlated with that at the ring C atoms (ΣC) (*R*^2^ = 0.9998, Figure 5a). If the atypical structures are omitted, then 66% (RO-DFT) (80%, U-DFT) of the partial spin at the ring C atoms (ΣC) is concentrated at the C(ipso) and C(p) atoms (Figure 5b, Table S4a–f).

The RO-DFT calculations suggest that the partial spin changes at C(ipso) and C(p) are weakly linearly correlated (Figure 5c), whereas no such correlation is predicted with the U-DFT approach. On the contrary, the U-DFT calculations show that the partial spin changes at C(o) and C(m) are correlated (Figure 5d), whereas the RO-DFT ones do not predict such a relationship. This difference may come from the fact that both signs of partial spin are allowed in the U-DFT approach, while only positive ones are present in the RO-DFT results (Table S4a–f). The other changes in the partial spin distributions found using the two approximations are analogous (Figure S4). 

Surprisingly, despite the correlation between NBO electron density and NBO spin density located at the specific C(o), C(m) or C(p) atom being either very weak or non-existent, a similar correlation taking into account all ring C atoms is not negligible (*R*^2^ = 0.884, Figure 5e). This is possible because overall charge and overall spin are separated between the ring and substituent, and constant charge/spin is populated at the ring H atoms. On the other hand, changes in the C(p) or the sum of the ortho and meta C atom charges with R are too weak to be statistically significant (Figure 4b). Interestingly, the partial charges at the C(o) and C(m) atoms are weakly linearly correlated (*R*^2^ = 0.63, Figure 4c).

The two U- and RO-DFT approximations reveal the same relationships between the partial charges in radical cations (Figure S3). Despite ca. 1.2 *e* being located at the ring H atoms, no partial spin is predicted to reside at these very atoms (Figure 5a, Table S4a–f). Thus, the spin at all substituent atoms is perfectly linearly correlated with that at the ring C atoms (ΣC) (*R*^2^ = 0.9998, Figure 5a). If the atypical structures are omitted, then 66% (RO-DFT) (80%, U-DFT) of the partial spin at the ring C atoms (ΣC) is concentrated at the C(ipso) and C(p) atoms (Figure 5b, Table S4a–f).

The RO-DFT calculations suggest that the partial spin changes at C(ipso) and C(p) are weakly linearly correlated (Figure 5c), whereas no such correlation is predicted with the U-DFT approach. On the contrary, the U-DFT calculations show that the partial spin changes at C(o) and C(m) are correlated (Figure 5d), whereas the RO-DFT ones do not predict such a relationship. This difference may come from the fact that both signs of partial spin are allowed in the U-DFT approach, while only positive ones are present in the RO-DFT results (Table S4a–f). 

The other changes in the partial spin distributions found using the two approximations are analogous (Figure S4). Surprisingly, despite the correlation between NBO electron density and NBO spin density located at the specific C(o), C(m) or C(p) atom being either very weak or non-existent, a similar correlation taking into account all ring C atoms is not negligible (*R*^2^ = 0.884, Figure 5e). This is possible because overall charge and overall spin are separated between the ring and substituent, and constant charge/spin is populated at the ring H atoms. 

Thus, the charge in the cation radicals, particularly in the ring, is mainly polarized between the C(ipso) atom and the substituent. Such anisotropic charge distribution makes the induction of the ring current in the π-electron system difficult, and therefore the NICS indices signalize the antiaromaticity of most of the rings (Figure 2b and Figure 3a). The location of partial spins mostly at the C(ipso) and C(p) atoms stabilizes the antiaromaticity of the systems.

### 2.5. The sEDA(D) and pEDA(D) Descriptors of Substituent Effect in Benzene Monocation Radicals

**Figure 6:**
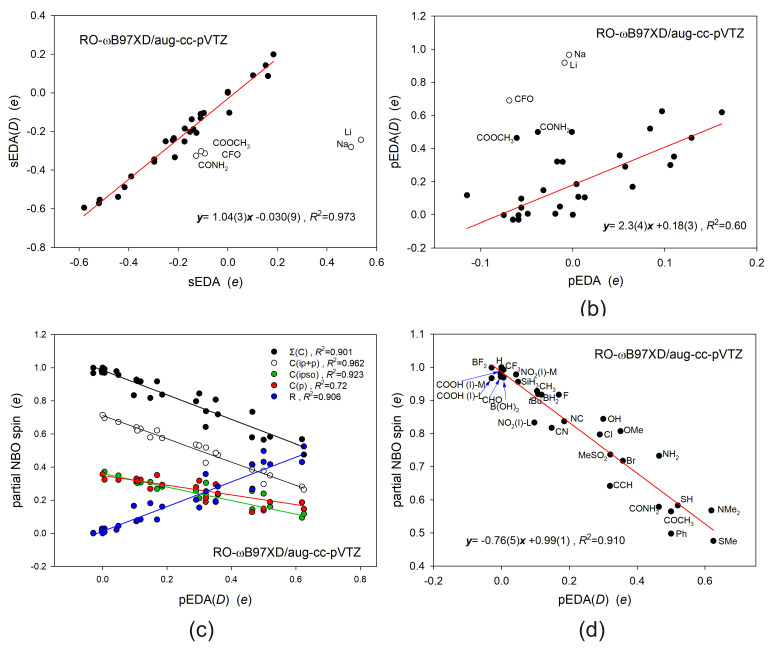
Linear regressions between the ground state sEDA (**a**) and pEDA (**b**) substituent effect descriptors and the reciprocal descriptors for the cation radicals. (**c**) Linear correlations between the pEDA(D) descriptor and spin densities at the sum of the ring C atoms (ΣC), and C(ipso), C(p), C(ipso+p) atoms, as well as at the substituent R, and (**d**) the correlation between the pEDA(D) descriptor and spin densities at the sum of the ring C atoms (ΣC) with the substituent type shown. The following regression lines were, respectively, obtained: y = −0.74(5)x + 0.98(1); y = −0.70(3)x + 0.71(1); y = −0.41(3)x + 0.36(1); y = −0.29(4)x + 0.35(1); y = −0.75(5)x + 0.01(1). All results were obtained with the RO-DFT method, but analogous relationships also adhere to the U-DFT data. In graphs (**c**,**d**), all atypical systems (forms (II) and (e)) were omitted.

The sEDA(D) and pEDA(D) descriptors revealing the influence on the σ- and π-valence electron systems in the ring (Table S5a,b, Figure 6 and Figure S5) were defined analogously to the previously constructed *sEDA* and *pEDA* (Equation (1)) type of descriptors [62,74,88,89,90]:

(1) $$\begin{array}{c} sEDA\left(D\right)=\sum_{i=1}^{6}\left(\sigma_{i}-\sigma_{ref}\right)\\ pEDA\left(D\right)=\sum_{i=1}^{6}\left(\pi_{i}-\pi_{ref}\right) \end{array}$$

where *σ_i_* and *π_i_* denote σ or *π* valence electron populations at the *i*-*th* carbon atom in the phenyl ring of the monosubstituted benzene in the monocation radical doublet *D* state, and superscript ref denotes the respective values in the reference unsubstituted benzene monocation radical in the *D* state.

Notice that the sEDA(*D*) and sEDA descriptors of the neutral molecules in the ground state are linearly correlated (*R*^2^ = 0.973, Figure 6a), whereas the analogous linear correlation between the pEDA(*D*) and pEDA descriptors is only weak (*R*^2^ = 0.60, Figure 6b). The substituent effect on the σ-valence orbitals has a short-range impact, while on the π-ones it has a long-range impact. Therefore, the strong correlation between the sEDA descriptors and the weak correlation between the pEDA ones mean that the effect with the C(ipso) atom is similar in the ground and cation radical states, but the substituent effect propagation through the π-electron structure is visibly different. This is not surprising if the charge distribution differences in the common and the quinone-like form of the ring are taken into account.

The outlying data are produced by the Li, Na, CFO, CONH_2_, and COCH_3_ derivatives (Figure 6a,b). For Li and Na systems, they are spread out because the metal atoms dissociate into C_6_H_5_^•^ and Li^+^ or Na^+^. The CFO, CONH_2_, and COCH_3_ derivatives differ from the other systems by quite a significant partial spin located at the substituents’ O atoms, while the spin at the linking C atom is null. The CONH_2_ and COCH_3_ are twisted out of the plane, whereas CFO is double bonded to the ring, and the spin is located at the O atom while vanishing at F. In the systems which are subject to the regular correlation trends, the spin at the R group is mainly focused at the linking X atom, even though in Ph it is symmetrically split into two rings, and in MeSO_2_ it is quite significant at the O atoms (Table S4a–d).

The partial spin at the sum of the ring C atoms (ΣC) and at the substituent R linearly changes with the pEDA(*D*) descriptor (Figure 6c), i.e., with the π-electron charge at the ring C atoms. This fact may seem obvious, but that is not necessarily so. Indeed, the almost perfect spin population on the π-electron structure with its simultaneous negligible presence on the σ-electron structure and ring H atoms could be not strictly preserved in some systems (Tables S5a and S6b, Figure S4g). Such spin location out of the π-electron system is observed in dissociated Li and Na derivatives, NO_2_ and COOH local minima, and CFO, COCH_3_, CONH_2_, and MeSO_2_ substituted systems (Figure S4g). Notice that the sum of the partial spin, C(ipso) + C(p) = C(ipso + p), correlates well with the pEDA(*D*) descriptor, and the contributions from the C(ipso) and C(p) partial spins are the very same (Figure 6c).

A closer inspection into the spin variation at (ΣC) with pEDA(*D*) reveals the substituents’ order, which is different from what we know from the ground state (Figure 6d). The COCH_3_ and CONH_2_ groups, which are π-electron withdrawing in the ground state (pEDA(CONH_2_) = −0.044, pEDA(COCH_3_) = −0.071) [62], fall among the most electron donating substituents such as NMe_2_ and SMe in the cation radicals. The CCH and CN groups, which are again π-electron withdrawing in the ground state (pEDA(CCH)= −0.011, pEDA(CN) = −0.032) [73], begin to act similarly to OH and OMe and be moderately π-electron donating (Figure 6d). The halogen atoms, which in the ground state have a similar slightly π-electron donating character (pEDA = 0.078, 0.062, 0.057, for F, Cl, and Br) [62], now donate much more, but in the inverse order (pEDA(*D*) = 0.158, 0.267, 0.358, for F, Cl, and Br, Table S6b). In the ground state, the BH_2_ group is the most π-electron withdrawing (pEDA = −0.142) [62], while in the cation radical it is π-electron donating (pEDA(*D*)= 0.118, Table S6b) and acts similarly to the F substituent (Figure 6d).

**Figure 7:**
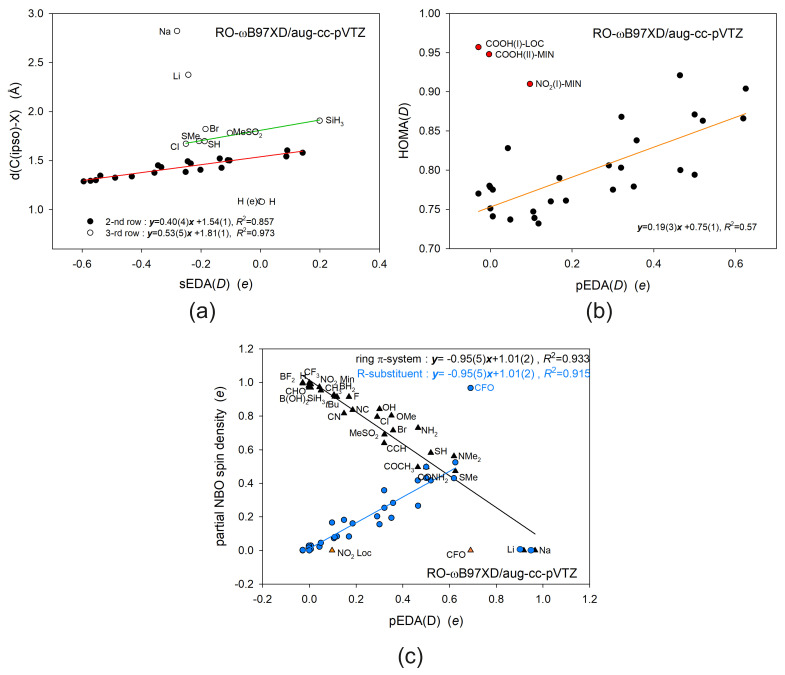
(**a**) Linear regression between the d(C(ipso)-X) in the cation radicals of the monosubstituted benzenes split into two straight lines: one for substituents in which the linking X atom belongs to the 2-nd row of the periodic table (red line) and the other in which it belongs to the 3-rd one (green). (**b**) Weak linear correlation between the HOMA(*D*) aromaticity index of the studied cation radicals and the pEDA(*D*) descriptor. (**c**) Linear correlations between the spin localized at the ring π-electron system (black) and at the substituent R (blue) and the pEDA(*D*) descriptor. Points in orange and for CFO substituent (blue) were omitted in correlations.

The sEDA(*D*) and pEDA(*D*) substituent effect descriptors correlate with some geometrical characteristics of the cation radicals in the *D* doublet state such as the d(C(ipso)-X) distance (Figure 6a) and HOMA(*D*) aromaticity index (Figure 7b). However, the correlation with d(C(ipso)-X) splits into two straight lines: one for substituents with the linking X atom belonging to the 2-nd row of the periodic table and the other in which it belongs to the 3-rd one (Figure 7a). The 1-st row H substituent is outlying, as well as the metal atoms, while the 4-th row Br substituent is accidentally placed at the regression line of the 3-rd row elements. We have already observed similar relationships for d(C(ipso)-X) distance in monosubstituted benzene derivatives in the first excited singlet state [73]. Due to the linear correlation between sEDA(*D*) and sEDA (Figure 7a), a similar correlation also holds for the d(C(ipso)-X) distance in radicals and the ground state sEDA descriptor (Figure S6a). A weak linear correlation occurs between the HOMA(*D*) aromaticity index and the pEDA(*D*) descriptor, showing that ca. 60% of geometrical aromaticity is connected to the charge present on the ring (Figure 7b). The limited strength of such a correlation is due to a specific bond alternation which is essential to aromaticity, though it cannot be included in a simple overall π-electron population in the ring. For U-DFT calculations, a similar relationship is obtained (Figure S6b).

There is also a counterintuitive linear correlation between the spin localized at the π-electron system of the ring and the pEDA(*D*) descriptor (Figure 7c). It demonstrates that the stronger the π-electron donating substituent, the smaller the spin population at the ring. However, the opposite direction of a linear correlation between the spin localized at the R substituent pEDA(*D*) shows that the stronger the π-electron donating substituent, the higher the spin population at the substituent (Figure 7c). Thus, in the benzene cation radicals, with the electron charge supplied to the ring π-electron system by the substituent, the spin flows in the opposite direction. This is connected with the relationship presented in Figure 5a, showing that the spin is split between R and the ring C atoms, and the fact that no spin is localized at the ring σ-electron system, except for Na and Li derivatives, which are dissociated, and the charge and spin remain mainly at the C(ipso) atom (Tables S4 and S6). In the CFO substituted cation radical, the spin is entirely located at the substituent, while ca. 0.7 *e* is shifted to the ring π-system.

Finally, let us mention that there are weak linear correlations between the spin localized at the π-electron system of the ring or pEDA(*D*) descriptor and the σ^•^ (para) descriptor of Dust and Arnold (Figure S7a,b, *R*^2^ = 0.68 and *R*^2^ = 0.54, respectively, the Shapiro–Wilk and Constant Variance tests are passed). Still, for the studied substituents, only some of the σ^•^ descriptors are known, and even those are only weakly correlated (Figure S7c, *R*^2^ ≈ 0.8). The σ^•^ descriptors were determined for the disubstituted systems and based on kinetic data. Thus, discrepancies between computational descriptors defined for monosubstituted benzene and experimental descriptors found for disubstituted benzenes are not very surprising.

## 3. Methods

Thirty monosubstituted cation radicals of benzene were considered. The functional groups used covered a wide range of effects on both σ and π valence electron systems of benzenes in the ground states and the first excited singlet states [62,83,84]. The structures were optimized using the unrestricted (U) [151,152] and the restricted open-shell (RO) [153,154] approximations. The ωB97XD98 DFT functional [155], inherently containing the dispersion correction, was combined with the aug-cc-pVTZ basis set [156,157]. The Gaussian 09, Rev. D1 suite of programs was applied [158]. The harmonic frequencies of all reported systems were positive, indicating the structures to be the true minima on PESs. Electron and spin populations were estimated using the NBO approach [91,92,93], as implemented in Gaussian 09. Correlation analysis was performed using the SigmaPlot 13 program [159].

The single reference RO-DFT and U-DFT methods used here have mutually verified their validity for benzene radicals. The U-DFT calculations are faster than RO-DFT ones, but they may produce spin contamination (artificial mixing of different spin states), whereas the RO-DFT computations are free from this error. In the unrestricted calculations, the spatial parts of α and β spin-orbitals are allowed to be different and the spin contamination provides wave functions which are not eigenfunctions of the total spin operator. The spin annihilation procedure reduces the size of the error in the unrestricted calculations, and then the expectation value of the total spin <S^2^> is close to S(S+1). On the other hand, the restricted open-shell calculations produce no spin contamination and give good wavefunctions and total energies, but the singly occupied orbital energies do not rigorously obey Koopman’s theorem [160].

In our previous study of the substituent effect in the first excited singlet and triplet states [73,74], we considered the ωB97XD functional together with the B3LYP with and without the D3 Grimme’s correction for dispersion forces and CAM-B3LYP. Yet, in the article we presented only the results obtained with the ωB97XD functional, because while performing similar to other functionals, it produced the lowest number of doubtful results [73,74]. The NMR properties were only able to be calculated at the U-DFT level [158].

## 4. Conclusions

The substituent effect in 30 monosubstituted benzene cation radicals was studied with the restricted open-shell (RO) and unrestricted (U) DFT methods using the ωB97XD/ag-cc-pVTZ level. The substituents were selected based on their ground state electron donating and/or withdrawing effects on both σ- and π-electron structure. They covered a wide range of effects. 

We found that most of the substituted benzene cation radicals adopt the compressed, quinoid-like, form (I) of the ring. For a few derivatives, a second compressed quinoid-like form (II) or an elongated one (e), which could coexist with form (I), was also predicted. However, two forms (II), being like two resonance structures to each other, in fact correspond to a single elongated form which cannot be automatically optimized computationally. Although we show computational arguments for the possible coexistence of two cation radical forms, we suggest that strong arguments for potential resonance isomers can only be justified at a higher level of theory. At this moment, it is reasonable to treat the prediction of different geometrical forms of radical cations obtained at the ωB97XD/ag-cc-pVTZ level with a certain reservation.

The comparison of our U- and RO-DFT computational and experimental literature ionization potential (IP) data suggests which form of benzene cation radical is more likely to exist. This is especially true when the U- and RO-DFT calculations concordantly suggest a certain IP. 

The geometrical and magnetic aromaticity calculated for the studied cation radicals, using the HOMA and NICS_ZZ_(1) indices, show that although the systems are structurally aromatic (HOMA > 0.7, i.e., higher than in most acenes), they are magnetically antiaromatic. They exhibit positive NICS_ZZ_(0) values of all rings, but some systems which are dissociated or not in the global minimum, and positive NICS_ZZ_(1) values except for SMe, NMe_2_, Ph, SH, CONH_2_, NH_2_, and COCH_3_ substituents. The benzene cation radicals example shows once more that aromaticity is a complex and multidimensional concept. 

The partial charges calculated using the NBO method and U- and RO-DFT approximations show that a constant amount of ca. 1.25 *e* is located at the five ring H atoms. The remaining charge is spread over the R substituent atoms and the ring C atoms (ΣC) and strongly linearly correlated (*R*^2^ = 0.987). In the ring, the charge at the C(ipso) atom varies the most and is linearly correlated with the charge at R (*R*^2^ = 0.86). The other changes at the ring C atoms with charge R are statistically insignificant, while the partial charges at the C(o) and C(m) atoms are weakly linearly correlated (*R*^2^ = 0.65). 

No partial spin is predicted to reside at the ring H atoms, and spin at all substituent atoms is perfectly linearly correlated with that at the ring C atoms (*R*^2^ = 0.9998). If the atypical structures are omitted, then 70% of spin (RO-DFT) is concentrated at the C(ipso) and C(p) atoms. The RO-DFT calculations suggest that the partial spin changes at C(ipso) and C(p) are linearly correlated. For partial spins, predictions found at the RO- and U- approximations differ a little, but data found using the RO-method seem to be in better agreement with chemical intuition.

We found the sEDA(*D*) and sEDA descriptors of the influence on the σ-electron system of the benzene moiety in the cation radicals and in neutral molecules in the ground state, respectively, to be linearly correlated. The effect on the σ-valence orbitals has a short-range impact and the correlation means that the effect at the C(ipso) atom is similar in the ground and cation radical states. The analogous linear correlation between the pEDA(*D*) and pEDA descriptors of the influence on the π-electron system is only weak. This shows that the propagation of the effect through the π-electron structure is visibly different in the ground and radical states due to differences in the π-electron charge distribution in the common and the quinone-like form of the ring. The partial spin at all ring C atoms (ΣC) and at the substituent R linearly changes with the pEDA(*D*) descriptor, i.e., with the π-electron charge at the ring C atoms. Additionally, the sum of the partial spin on C(ipso) and C(p) atoms correlates well with the pEDA(*D*) descriptor, and the contributions from the C(ipso) and C(p) partial spins are essentially the same. Interestingly, the ΣC spin variation with pEDA(*D*) reveals that the substituents’ order in the cation radical state is different from what we know from the ground state. 

The sEDA(*D*) substituent effect descriptors correlate with d(C(ipso)-X) distance in cation radicals, while the pEDA(*D*) descriptor weakly correlates with the HOMA(*D*) aromaticity index. A counterintuitive linear correlation between the spin localized at the π-electron system of the ring and the pEDA(*D*) descriptor shows that the stronger the π-electron donating substituent, the smaller the spin population at the ring. This occurs because in the benzene cation radicals, with the electron charge supplied to the ring π-electron system by the substituent, the spin flows in the opposite direction. This is connected with the relationship showing that spin is split between R and the ring C atoms, and the fact that no spin is localized at the ring σ-electron system.

## Data Availability

Not applicable.

## Supplementary Materials

The following are available online at https://www.mdpi.com/article/10.3390/ijms22136936/s1.
