# Supplementary material for: Substituent Effect in the Cation Radicals of Monosubstituted Benzenes
Source: Int J Mol Sci. 2021 Jun 28;22(13):6936. doi: 10.3390/ijms22136936 (PMC8269098; doi:10.3390/ijms22136936)
Supplement: Supplementary file 1 [file ijms-22-06936-s001.zip › ijms-1244481-supplementary.pdf]

Supplementary Information File to: Substituent Effect in the Cation Radicals of Monosubstituted Benzenes

Jan Cz. Dobrowolski,[*,1,2] Wojciech M. Dudek,[2] Grażyna Karpińska,[1] Anna Baraniak[1]

[1]National Medicines Institute, 30/34 Chełmska Street, 00-725 Warsaw, Poland

[2]Institute of Nuclear Chemistry and Technology, 16 Dorodna Street, 03-195 Warsaw, Poland

	Content	Page
**text**	Supplementary information about the studies on the characterization of radicals:	2
**Ref.**	SI References	3
**Figure S1.**	energies of the monocation radicals	5
**Figure S2.**	magnetic aromaticity	6
**Figure S3.**	partial NBO charges	7
**Figure S4.**	partial NBO spins	8
**Figure S5.**	sEDA and pEDA substituent effect descriptors	9
**Figure S6.**	the d(C(ipso)-X) and HOMA(*D*) correlations	9
**Figure S7.**	Weak correlations with the $\sigma^{\bullet}$ (para) descriptor of Dust and Arnold	10
**Table S1a.**	U-DFT The total, zero-point vibrational, and Gibbs free energy	11
**Table S1b.**	RO-DFT The total, zero-point vibrational, and Gibbs free energy	12
**Table S1c.**	Ground State R-DFT The total, zero-point vibrational, and Gibbs free energy	13
**Table S1d.**	U-DFT The total, zero-point vibrational, and Gibbs free energy in the geometry fixed as in the ground state	14
**Table S1e.**	RO-DFT The total, zero-point vibrational, and Gibbs free energy in the geometry fixed as in the ground state	15
**Table S1f.**	the ionization energies without the change of ground state energy and the relaxation energies, calculated using the U- and RO-DFT	16
**Table S2a.**	U- and RO-DFT CC and C(ipso)-R bond distances and HOMA aromaticity indices	17
**Table S2b.**	Juxtaposition of the HOMA and NICSzz aromaticity parameters	18
**Table S3a.**	U-DFT The NBO partial charges	19
**Table S3b.**	U-DFT The ordered and summed NBO partial charges	20
**Table S3c.**	RO-DFT The NBO partial charges	21
**Table S3d.**	RO-DFT The ordered and summed NBO partial charges	22
**Table S4a.**	U-DFT The NBO partial spins	23
**Table S4b.**	U-DFT The ordered and summed NBO partial spins	24
**Table S4c.**	RO-DFT The NBO partial spins	25
**Table S4d.**	RO-DFT The ordered and summed NBO partial spins	26
**Table S5a.**	U-DFT The NBO valence ring orbital electron and spin populations	27
**Table S5b.**	RO-DFT The NBO valence ring orbital electron and spin populations	28
**Table S6.**	The $\sigma^{\bullet}$ values for the substituents considered in this study according to	29

	Hansch and Gao.	

Supplementary information about the studies on the characterization of radicals:

Several other descriptors were designed to account for the substituent effect on radicals:[62] The Alfrey and Price Q and e[73] were developed in 1947 for radical polymerization and the Q parameter evaluated the radical stabilization, and the e, the polar factors; the resonance E_D value of Sakurai, Hayasaki, and Hosomi correlated well with log(Q);[74] the Ito et al. τ parameters described the conjugative effects in the ortho and para positions;[75] the Fisher-Meierhoefer $\sigma^\bullet$ scale was obtained based on the kinetics of the N-bromosuccinimide bromination of 4-substituted 3-cyanotoluenes;[76] the Jackson $\sigma^\bullet$ descriptor was derived from decomposition of substituted dibenzylmercury compounds;[77,78] and the Adam et al. EPR zero-field splitting D parameter and $\Delta D = D_H - D_X$ scale were obtained based on cyclopentane-1,3-diyl diradical.[79,80]

With the proliferation of the quantum chemical methodology and computational power, the usefulness of the theoretical parameters in modeling the substituent effect on radicals' reactions was demonstrated in a number of studies. In 1981, semiempirical Hückel calculations of radical stabilization and coupling constants changes in series of substituted benzyl radicals were performed.[81] In 1996, the Adam's D parameter was shown to correlate well with the one constructed based on interspin distances and spin densities calculated at the semiempirical PM3 level.[82] The bond dissociation energies (BDE) obtained from AM1 calculated isodesmic reactions were also proved to correlate well with the Hammett σ constant and to thoroughly describe the homolytic cleavage of the benzylic bonds.[83] Also in 1996, Wu and Lai[84] modeled the BLYP/6-31G* calculated change in spin density and in BDE, in a series of para substituted phenols anisoles, using $\sigma_{JJ}^\bullet$ of Jiang and Ji. The spin delocalization was maximized with the π-donor ligands. The correlation found was weaker than that observed between $\sigma p+$ and charge density. Interestingly, although almost all substituents in para position stabilized the phenoxy radical by spin delocalization, the electron-donating groups stabilized but the electron-withdrawing groups destabilized the phenoxy radical by the polar effect i.e., charge redistribution. The polar and radical contributions of the substituent effect on the phenols OH bond dissociation energy were studied with computational methods by Brinck, Haeberlein, and Jonsson, who showed that the electron-donating substituents destabilized the phenol and, less effectively, stabilized the radical. On the other hand, the electron-withdrawing substituents stabilized the phenol and yielded irregular radical effects. The methodology employed in this work could probably be used to differentiate between polar and radical substituent effects in other systems as well.[85] The combined DFT/B3LYP and AM1 semiempirical studies performed in 1999 by Pratt, Wright, and Ingold[86] demonstrated precise agreement between the calculated and measured radical constants and leaded to the conclusion that the magnitude of the effects in para substituted benzyl halides does not depend on the electronegativity of the methyl halides substituents. The radical stabilization energies determined based on the isodesmic reaction between para substituted benzylic radicals with toluene calculated at the DFT/B3LYP/6-31G* level by Creary et al.[87] correlated well with the rates of the methylenecyclopropane rearrangement. This indicated the substantial spin delocalization produced by substituents on the para-position of the benzylic radical. The RX-H (X=C, N, O, S) dissociation thermochemistry of different reactions types was studied with various DFT approaches combined with series of basis sets by DiLabio et al. [88] ena-

bling, inter alia, the determination of redox potentials and energies of the H-atom transfer radical reactions. Several strong correlations between bond dissociation energy and the σ^+ constant, as well as the standard reduction potential, were shown.[88]

The UB3LYP/6-31G* calculated substituent effect on the radical stabilization energy of para-substituted phenylacetonitrile radicals showed significant correlation with the σ^+ constants and the calculated hyperfine coupling constants were found to agree very well with the experimental ones.[89] Moreover, the calculated spin-derived parameters for four substituents correlated linearly with the calculated relative phenyl ring charges.[89] In 2002, Pratt et al. presented strong correlations between σ_P^+ constants and bond dissociation energies or enthalpies, or radical stabilization energies calculated at the RO-B3LYP/6-311+G(2d,2p)//AM1/AM1 level for aromatic amines.[90] The details of such correlations were also discussed later.[91] Liu et al. considered presence of the Hammett (correlating with the σ constant) and non-Hammett effects in series of para-substituted benzenes calculated at the B3LYP/6-311++G(2d,2p) level. [92] They suggested that the nonpolar resonance effect is orthogonal to the previously documented substituent effects and to be describable by $\sigma^\bullet$ descriptors. The analysis of the B3LYP/6-31++G** calculated $\sigma^\bullet_\alpha$ descriptor (estimated based on calculated proton hyperfine coupling constants according to Dust and Arnold expression[67]) for six para-substituted benzyl radicals showed excellent agreement with the experimental values and proved that the bond dissociation enthalpy depended solely on radical stabilization resulted from the spin density delocalisation.[93] The study also revealed an interesting order of substituents stabilising the radicals: NMe_2 > CHO > NH_2 > NO_2 > COOH = CN > OH > OCH_3 > CH_3 > CF_3 where pairs of substituents like (NMe_2, CHO) or (CN, OH), are neighboring and despite the non-radicals, they exhibit the opposite π-electron effect. Important finding was reported in 2008 by Zavitsas who provided a scale of radical destabilization energies with a common zero for all species based on Pauling's electronegativity equation.[94]

The reasons why the charge and spin distributions in substituted phenoxy radicals can be uncorrelated were studied by Fehir and McCusker.[95] They demonstrated that polarization of charge density varies with the inductive and/or resonance effects expressed by classical σ and σ+ constants, whereas the spin density was insufficiently described by known Hammett-type spin delocalization constants. It appeared that the polarization of spin density was significantly correlated with the α(SOMO-SUMO) gap. The reason for different spin and charge polarization was suggested to stem from the fact that spin density is carried by the unpaired electron occupying SOMO, whereas charge density reflected the behavior of all electrons in the system. A correlation between decreasing BDE and the presence of π-donors was noted, suggesting that stabilization of the radical is associated with increased spin delocalization of the odd electron.

SI References

(62) Cherkasov, A. 'Inductive' Descriptors: 10 Successful Years in QSAR. *Curr. Comput. Aided Drug Des*. **2005**, *1*, 21-42.

(63) Jaffé, A Reëxamination of the Hammett Equation. H. H. *Chem. Rev*. **1953**, *53*, 191-261.

(64) Yukawa Y, Tsuno Y. Resonance Effect in Hammett Relationship. II. Sigma Constants in Electrophilic Reactions and their Intercorrelation. *Bull. Chem. Soc. Jpn*. **1959**, *32*, 965-971.

(64) Stock, L. M.; Brown, H. C. A Quantitative Treatment of Directive Effects in Aromatic Substitution. in *Advances in Physical Organic Chemistry*, Gold, V. (Ed.), Academic Press, Inc., London, **1963**, pp. 35-154.

(65) Yamamoto, T. The Effect of Substituents in Radical Reactions: Reactivities of Substituted Cumenes to Attack by the Polystyryl Radical. *Bull. Chem. Soc. Jpn.* **1967**, *40*, 642-645. and references therein.
(66) Dust, J. M.; Arnold, D. R. Substituent effects on benzyl radical ESR hyperfine coupling constants. The $\sigma_{\alpha}^{\bullet}$ scale based upon spin delocalization. *J. Am. Chem. Soc.* **1983**, *105*, 1221–1227.
(67) Arnold, D. R. The Effect of Substituents on Benzylic Radical ESR Hyperfine Coupling Constants. The $\sigma^{\bullet}$ Scale Based Upon Spin Delocalization. pp. 171-188 in Substituent Effects in Radical Chemistry. Viehe, H. G.; Janousek, Z.; Merényi, R. (Eds.), NATO ASI Series, Vol. 189, Springer, 1986.
(68) Creary, X. Rearrangement of 2-aryl-3,3-dimethylmethylenecyclopropanes. Substituent effects on a nonpolar radical-like transition state. *J. Org. Chem*. **1980**, *45*, 280–284.
(69) Creary, X.; Mehrsheikh-Mohammadi, M. E.; McDonald, S. Methylenecyclopropane rearrangement as a probe for free radical substituent effects. $\sigma^{\bullet}$ Values for commonly encountered conjugating and organometallic groups. *J. Org. Chem.* **1987**, *52*, 3254–3263.
(70) Jiang, X.; Ji, G. A self-consistent and cross-checked scale of spin-delocalization substituent constants, the $\sigma^{\bullet}_{JJ}$ scale. *J. Org. Chem.* **1992**, *57*, 6051–6056.
(71) Héberger, K.; Lopata, A.; Jászberényi, J. Cs. Separation of polar and enthalpy effects in radical addition reactions using polar (σ) and radical ($\sigma^{\bullet}$) sigma scales. *J. Phys. Org. Chem*. **2000**, *13*, 151–156.
(72) Alfrey, Jr, T.; Price, C.C. Relative Reactivities in Vinyl Copolymerization. *J. Polym. Sci*. **1947**, *2*, 101-106.
(73) Sakurai, H.; Hayasaki, S.; Hosomi, A. Relative Reactivities in the Addition of Free Trichloromethyl Radicals to Substituted Styrenes. An Attempt to Separate Polar and Resonance Effects. *Bull. Chem. Soc. Jpn*. **1971**, *44*, 1945-1949.
(74) Ito, R.; Migita, T.; Morikawa, N.; Simamura, O. Influence of substituent groups in the arylation of substituted benzenes by aryl radicals derived from *p*-substituted N-nitrosoacetanilides. *Tetrahedron*, **1965**, *21*, 955-961.
(75) Fisher, T.H.; Meierhoefer, A.W. Substituent Effects in Free-Radical Reactions. A Study of 4-Substituted 3-Cyanobenzyl Free Radicals. *J. Org. Chem.* **1978**, *43*, 224-228.
(76) Dinçtürk, S.; Jackson, R. A.; Townson, M.; Ağirbaş, H.; Billingham, N. C.; March, G. Free radical reactions in solution. Part 6. Thermal decomposition of substituted dibenzyl mercurials in solution. An improved $\sigma^{\bullet}$ scale. *J. Chem. Soc., Perkin Trans. 2*, **1981**, 1121–1126.
(77) Dinçtürk, S.; Jackson, R. A. Free radical reactions in solution. Part 7. Substituent effects on free radical reactions: comparison of the $\sigma^{\bullet}$ scale with other measures of radical stabilization. *J. Chem. Soc., Perkin Trans. 2*, **1981**, 1127–1131.
(78) Adam, W.; Kita, F.; Harrer, H. M.; Nau, W. M.; Zipf, R. The *D* Parameter (EPR Zero-Field Splitting) of Localized 1,3-Cyclopentanediyl Triplet Diradicals as a Measure of Electronic Substituent Effects on the Spin Densities in *Para*-Substituted Benzyl-Type Radicals. *J. Org. Chem.* **1996**, *61*, 7056–7065.
(79) Adam, W.; Harrer, H. M.; Kita, F.; Nau, W. M. Localized triplet diradicals as a probe for electronic substituent effects in benzyl-type radicals: The Δ*D* scale. *Pure Appl. Chem.* **1997**, *69*, 91–96.
(80) Dinçtürk, S.; Jackson, R. A.; Townson, M.; Ağirbaş, H.; Billingham, N. C.; March, G. Free radical reactions in solution. Part 6. Thermal decomposition of substituted dibenzyl mercurials in solution. An improved $\sigma^{\bullet}$ scale. *J. Chem. Soc., Perkin Trans. 2*, **1981**, 1121–1126.
(81) Adam, W.; Harrer, H. M.; Heidenfelder, T.; Kammel, T.; Kita, F.; Nau, W. M.; Sahin, C. The *D* parameter (zero-field splitting) as a direct measure of structural and electronic effects in localized triplet 1,3-diradicals. *J. Chem. Soc., Perkin Trans. 2*, **1996**, 2085-2089.
(82) Nau, W. M. Computational Assessment of Polar Ground-State Effects on the Bond Dissociation Energies of Benzylic and Related Bonds. *J. Org. Chem.* **1996**, *61*, 8312–8314.
(83) Wu, Y.-D.; Lai, D. K. W. A Density Functional Study of Substituent Effects on the O–H and O−CH3 Bond Dissociation Energies in Phenol and Anisole. *J. Org. Chem*. **1996**, *61*, 7904–7910.

(84) Brinck, T.; Haeberlein, M.; Jonsson, M. A Computational Analysis of Substituent Effects on the O−H Bond Dissociation Energy in Phenols: Polar Versus Radical Effects. *J. Am. Chem. Soc.* **1997**, *119*, 4239–4244.

(85) Pratt, D. A.; Wright, J. S.; Ingold, K. U. Theoretical Study of Carbon−Halogen Bond Dissociation Enthalpies of Substituted Benzyl Halides. How Important Are Polar Effects? *J. Am. Chem. Soc.* **1999**, *121*, 4877-4882.

(86) Creary, X.; Engel, P. S.; Kavaluskas, N.; Pan, L.; Wolf, A. Methylenecyclopropane Rearrangement as a Probe for Free Radical Substituent Effects. $\sigma^\bullet$ Values for Potent Radical-Stabilizing Nitrogen-Containing Substituents. *J. Org. Chem.* **1999**, *64*, 5634–5643.

(87) DiLabio, G. A.; Pratt, D. A.; LoFaro, A. D.; Wright, J. S. Theoretical Study of X−H Bond Energetics (X = C, N, O, S): Application to Substituent Effects, Gas Phase Acidities, and Redox Potentials. *J. Phys. Chem. A*, **1999**, *103*, 1653–1661.

(88) Wen, Z.; Li, Z.; Shang, Z.; Cheng, J.-P. On the Direction and Magnitude of Radical Substituent Effects: The Role of Polar Interaction on Thermodynamic Stabilities of Benzylic C−H Bonds and Related Carbon Radicals. *J. Org. Chem.* **2001**, *66*, 1466–1472.

(89) Pratt, D. A.; DiLabio, G. A.; Valgimigli, L.; Pedulli, G. F.; Ingold, K. U. Substituent Effects on the Bond Dissociation Enthalpies of Aromatic Amines. *J. Am. Chem. Soc.* **2002**, *124*, 11085-11092.

(90) Pratt, D. A.; DiLabio, G. A.; Mulder, P.; Ingold, K. U. Bond Strengths of Toluenes, Anilines, and Phenols: To Hammett or Not. Acc. Chem. Res. **2004**, *37*, 334–340.

(91) Liu, L.; Cheng, Y.-H.; Fu, Y.; Chen, R.; Guo, Q.-X. The Nonpolar Resonance Effects and the Non-Hammett Behaviors. *J. Chem. Inf. Comp. Sci.* **2002**, *42*, 1164–1170.

(92) Singh, N. K.; Popelier, P. L. A.; O'Malley, P. J. Substituent effects on the stability of para substituted benzyl radicals. *Chem. Phys. Lett*. **2006**, *426*, 219–221.

(93) Zavitsas, A. A. A Single Universal Scale of Radical Stabilization Energies Does Not Exist: Global Bond Dissociation Energies and Radical Thermochemistries Are Described by Combining Two Universal Scales. *J. Org. Chem*. **2008**, *73*, 9022–9026.

(94) Fehir, Jr., R. J.; McCusker, J. K. Differential Polarization of Spin and Charge Density in Substituted Phenoxy Radicals. *J. Phys. Chem. A* **2009**, *113*, 9249–9260.

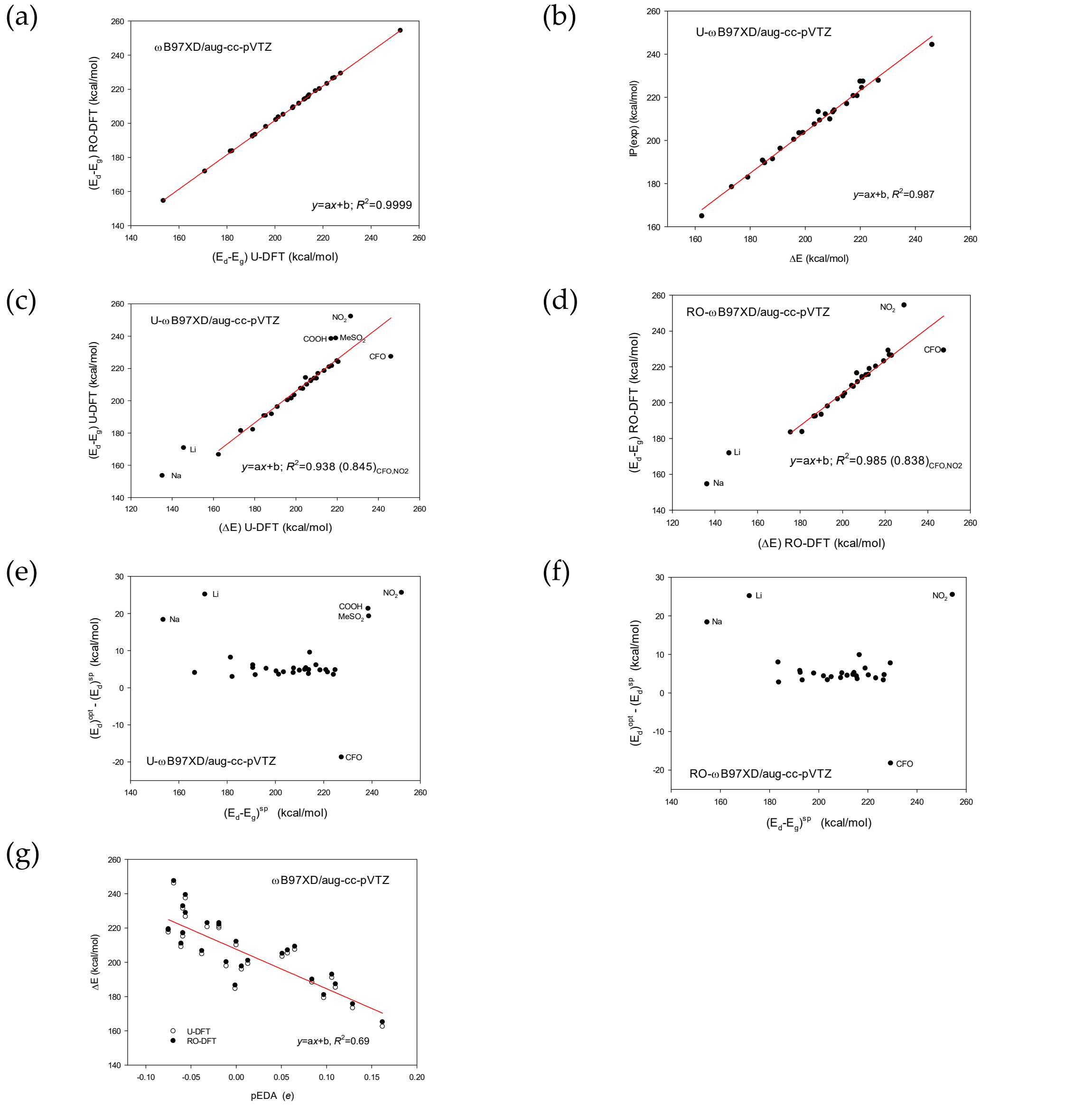

Figure S1. (a) The relative single point energies of the monocation radicals (E_d-E_g) (kcal/mol) calculated in the geometries of the ground singlet state. (b) Linear correlations between experimental ionization energies and the relative values of total energies (ΔE, kcal/mol) of monosubstituted benzene monocation radicals calculated using U-DFT approximations and the ωB97XD/aug-cc-pVTZ level, referred to the energies of the molecules in the ground singlet state calculated at the same level. Correlation between the total energy differences of the radicals in the ground singlet geometry (E_d-E_g) and the ones of the optimized singlet state structures: (c) U- and (d) RO-DFT approximations. The data for Li and Na substituents were omitted because of the metal ion dissociation. The squares of the correlations coefficients, R^2, are given for the set with and without the outlying points. Comparison between ionization energy and relaxation energy (see text) calculated with U-DFT (e) and RO-DFT (f) methods. (g) Weak linear correlations between relative values of total energies (ΔE, kcal/mol) and the pEDA substituent descriptor reflecting substituent influence on the benzene π-electron system in the ground state.

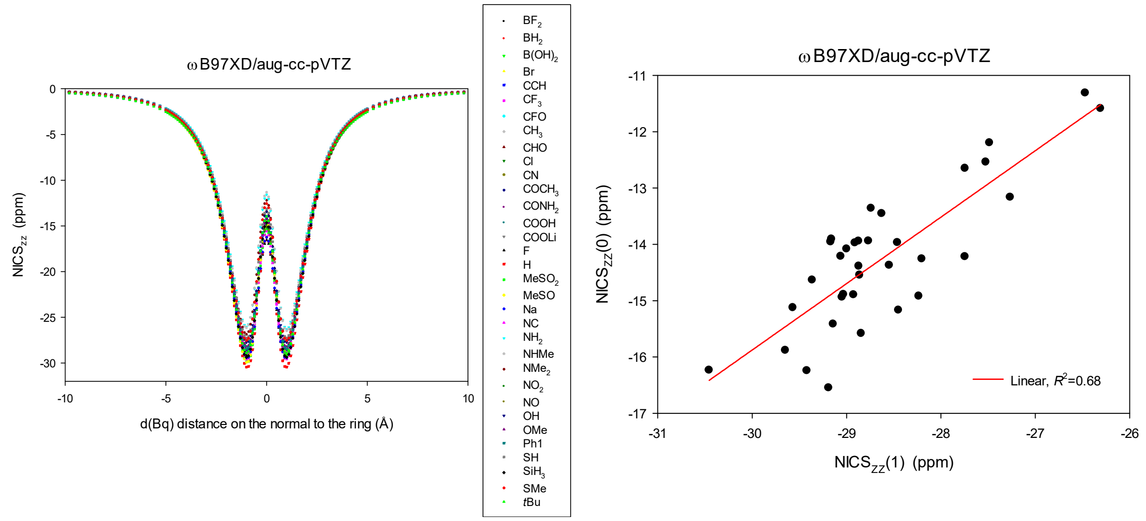

(a) (b)

Figure S2. (a) The NICSzz scans against sample point distance d(Bq) from on the normal to the mono-substituted benzene ring passing through the ring center which is placed in the origin. The molecule is located in the XY plane. (b) Weak linear correlation between the NICSzz(1) and NICSzz(0) indices calculated for the monosubstituted benzenes in the ground state. The calculations were performed at the ωB97XD/aug-cc-pVTZ level.

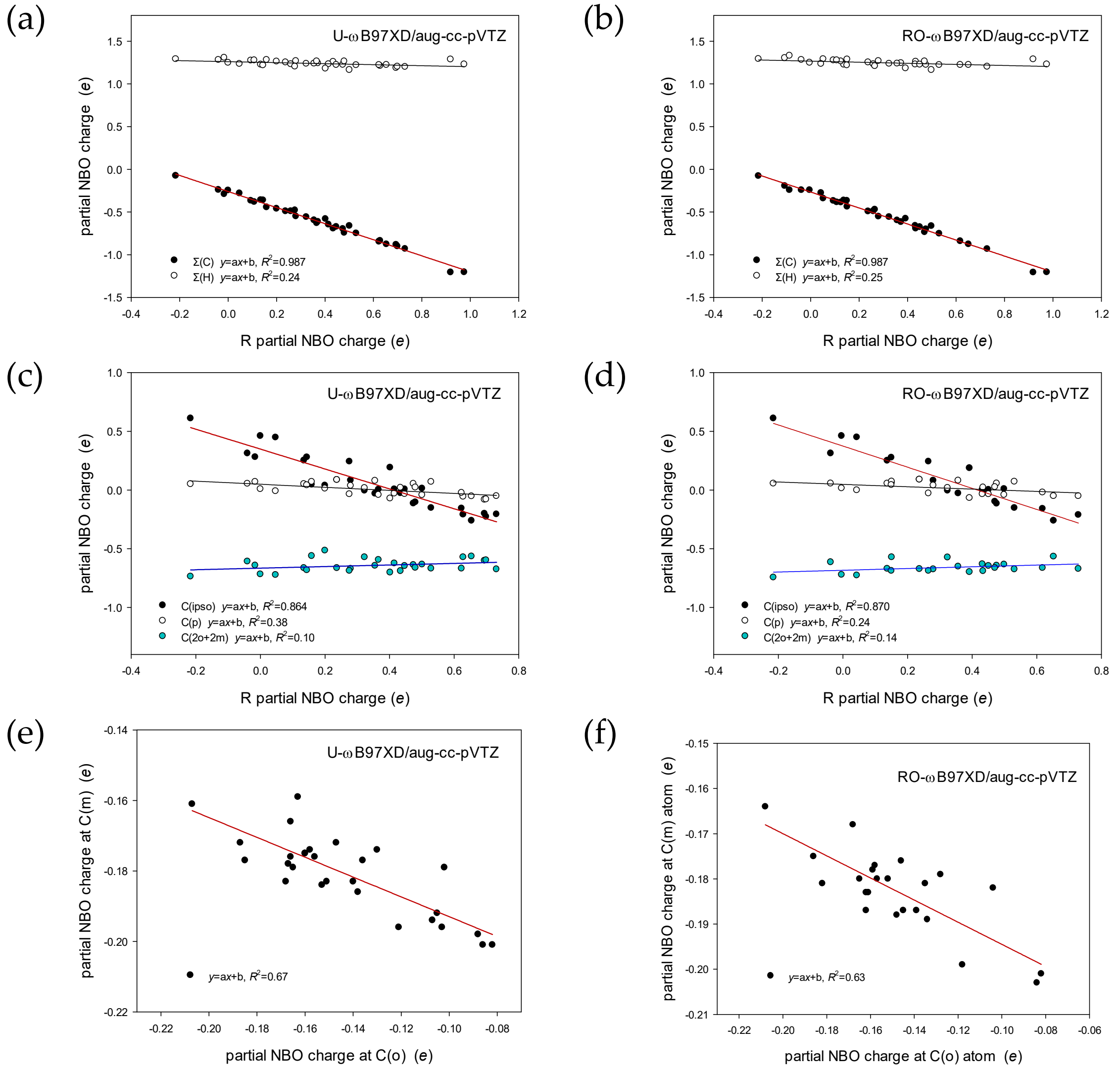

Figure S3. Linear changes of the sum of the partial NBO charges at the ring C atoms, Σ(C), and lack of similar changes of the sum of charges at the ring H atoms, Σ(H), in the monosubstituted benzene cation radicals with the change of the charge at all R substituent atoms calculated using the U-DFT (a) and RO-DFT (b) methods. Linear changes of the partial NBO charge at the C(ipso) atom, and essentially, a lack of similar changes at the C(p), or the sum of the *ortho* and *meta* C atoms in the monosubstituted benzene cation radicals with the change of the charge at all R substituent atoms calculated using the U-DFT (c) and RO-DFT (d) methods. In graphs (c)-(f) all atypical systems (forms (II) and (e)) were omitted.

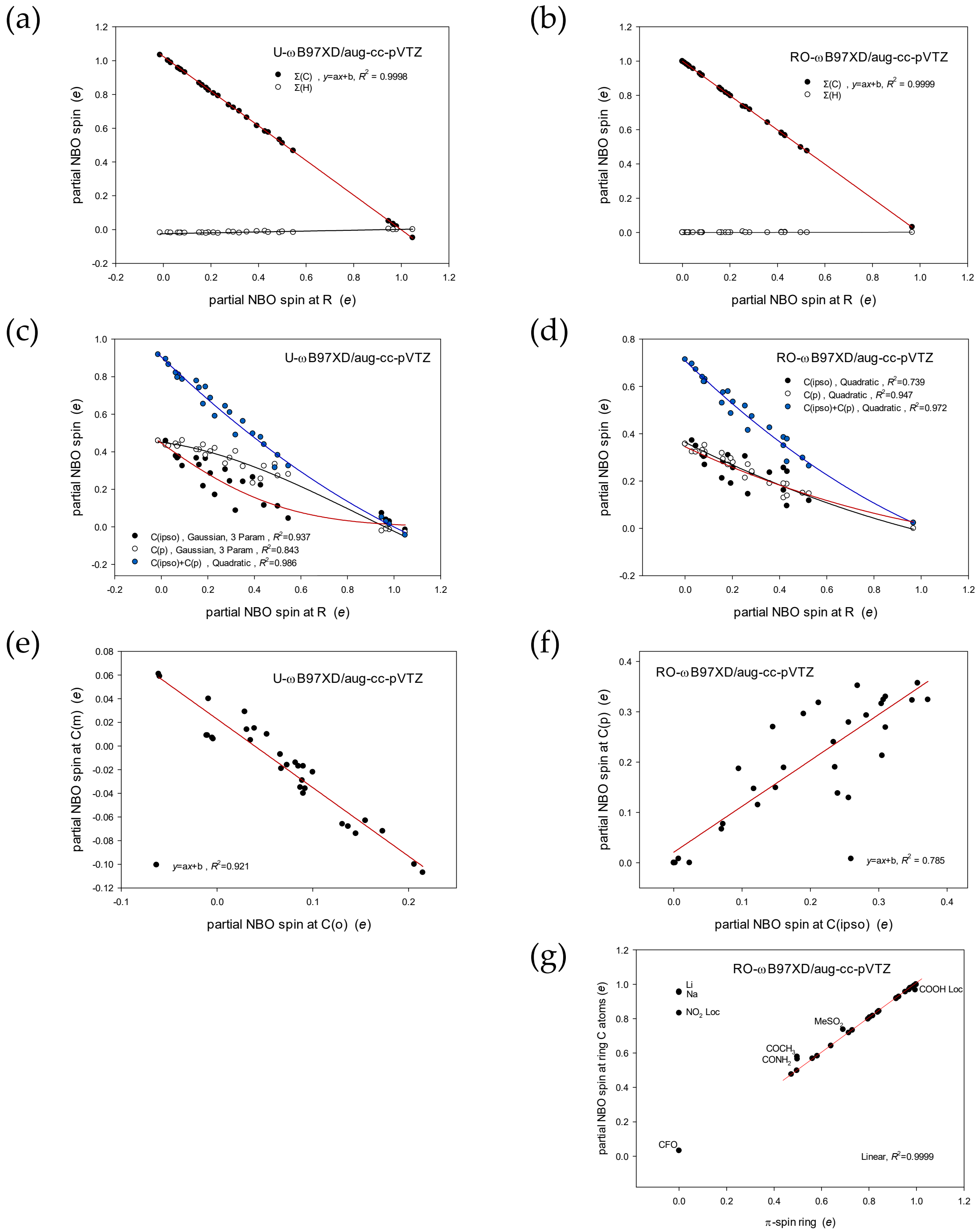

Figure S4. Linear changes of the sum of the partial NBO spins at the ring C atoms, Σ(C), and the lack of similar changes at the sum of ring H atoms, Σ(H), in the monosubstituted benzene cation radicals with the partial spin at all R substituent atoms calculated using the U-DFT (a) and RO-DFT (b) methods. Slightly non linear correlations of the partial NBO spin at the sum of C(ipso) and C(p) atoms with the partial spin at all R substituent atoms, and similar but weaker correlations for separate C(ipso) and C(p) atoms (c) and (d). A significant linear correlation between partial spin located at C(o) and C(m) atoms calculated using U-DFT method (e) and a fair linear correlation between partial spin located at C(ipso) and C(p) atoms calculated using RO-DFT method (f). In graphs (c)-(f) all atypical systems (forms (II) and (e)) were omitted.Excellent correlation between spin located at the π-electron system of the ring

and the partial spin located at thering C-atoms. The outlyers have either significant spin located at σ-elektron system (Li, Na) or at some atom(s) of the substituent.

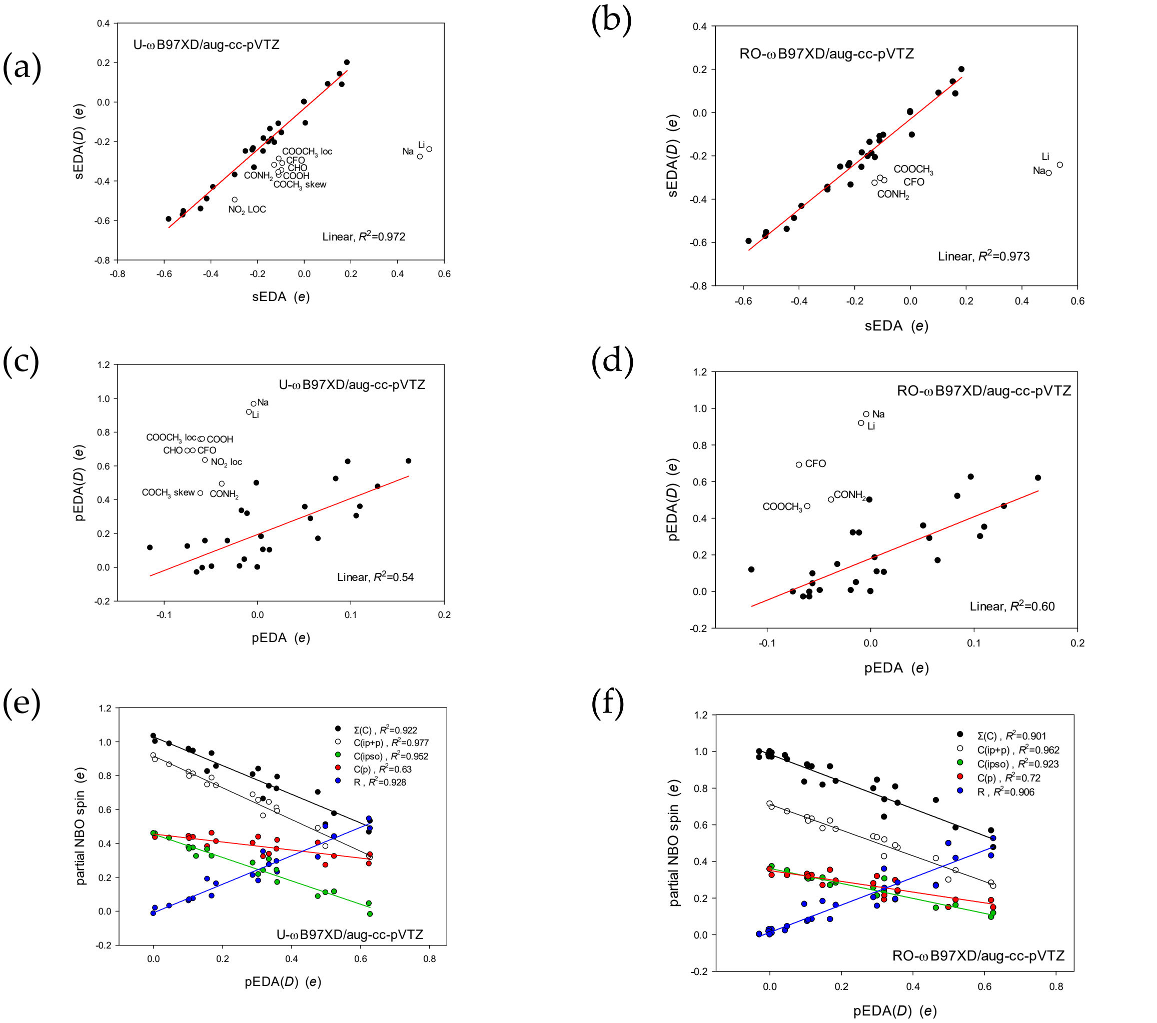

Figure S5. Linear regressions between the ground state sEDA and pEDA substituent effect descriptors and the reciprocal descriptors for the cation radicals obtained with the U-DFT (a) and (c) and RO-DFT (b) and (d) methods. Linear correlations between the pEDA(D) descriptor and spin densities at the sum of the ring C atoms (ΣC), and C(ipso), C(p), C(ipso+p) atoms, as well as at the substituent R calculated with the U-DFT (e) and RO-DFT (f) methods. In graphs (c)-(f) all atypical systems (forms (II) and (e)) were omitted.

(a)

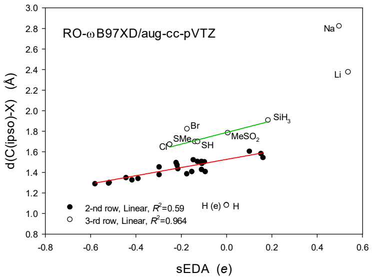

(b)

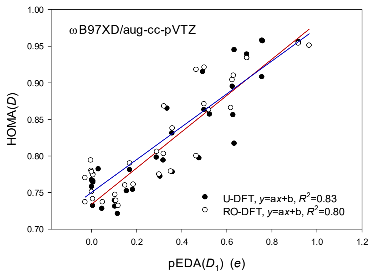

Figure S6. (a) Linear regression between the d(C(ipso)-X) in the cation radicals of the monosubstituted benzenes and the ground state sEDA descriptor. The regression also splits into two straight lines: one for substituents in which the linking X-atom belongs to the 2-nd row of the periodic table (red line) and the other in which it belongs to the 3-rd one (green). (b) Weak linear correlations between the HOMA(D) aromaticity index of the studied cation radicals and the pEDA(D) descriptor calculated with U- and RO-DFT methods.

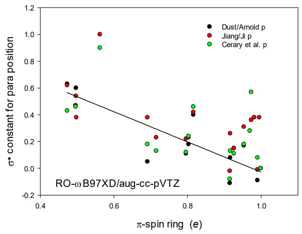

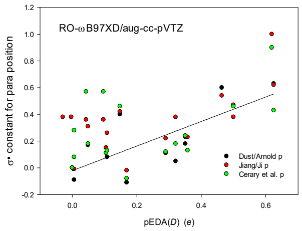

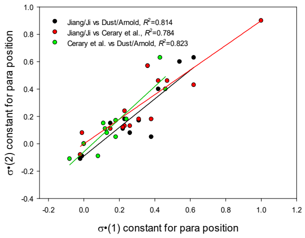

(a) (b) (c)

Figure S7. (a) Weak linear regression between spin localized at the π-electron system of the ring and the $\sigma^{\bullet}$ (para) descriptor of Dust and Arnold. (b) Weak linear regression between the pEDA(D) descriptor and the $\sigma^{\bullet}$ (para) descriptor of Dust and Arnold. (c) Mutual correlations between different $\sigma^{\bullet}$ (para) descriptors for the set of studied compounds (Table S6).

Table S1a. The total, zero-point vibrational, and Gibbs free energy (hartree) for monosubstituted benzene monocation radicals calculated using the U-DFT method with the ωB97XD functional and the aug-cc-pVTZ basis set, and relative values referred to energies of the molecules in the ground singlet state calculated at the R-ωB97XD/aug-cc-pVTZ level.

substituent	U-ωB97XD					
	E	ΔE	ZPE	ΔZPE	G	ΔG
BF_2 (II)	-456.000212	216.2	-455.902343	213.9	-455.936587	212.9
BH_2	-257.343861	210.8	-257.234666	208.9	-257.265515	207.9
$B(OH)_2$	-407.961511	202.4	-407.837722	201.0	-407.872118	200.0
Br	-2805.580080	203.4	-2805.489749	202.8	-2805.521518	202.2
CCH	-308.069418	197.8	-307.959942	197.2	-307.991328	196.5
CF_3 (II)	-568.984245	220.0	-568.880518	218.4	-568.915425	218.3
CFO	-444.458717	246.1	-444.355648	245.7	-444.387869	245.6
CH_3	-271.245798	199.2	-271.118914	198.1	-271.150542	198.0
CHO (I) LOC	-345.223957	218.9	-345.115123	217.8	-345.146337	217.4
CHO (I) MIN skew	-345.226292	217.5	-345.118733	215.5	-345.150925	214.5
Cl	-691.534589	205.3	-691.443897	204.7	-691.474613	204.1
CN	-324.130704	220.6	-324.032732	219.3	-324.064139	218.5
$COCH_3$ (I) LOC	-384.562669	210.6	-384.425447	209.5	-384.458954	209.0
$COCH_3$ (I) MIN	-384.565130	209.0	-384.428193	207.8	-384.462368	206.9
$CONH_2$	-400.637325	204.8	-400.543503	182.9	-400.509641	224.5
COOH (II) MIN	-420.494894	215.1	-420.380771	213.4	-420.414182	212.6
COOH (I) LOC	-420.468920	231.4	-420.353080	230.8	-420.385201	230.8
F	-331.164681	207.4	-331.072780	206.6	-331.102604	206.0
H	-231.909106	210.1	-231.810435	208.4	-231.839036	207.3
Li	-238.903126	145.6	-238.812881	145.8	-238.842388	145.9
$MeSO_2$	-819.842985	219.4	-819.705557	217.9	-819.742097	216.9
Na	-393.676873	135.2	-393.587499	135.5	-393.618869	135.4
NC	-324.108883	213.8	-324.011295	212.7	-324.042775	212.0
NH_2	-287.332595	173.3	-287.214075	173.6	-287.243834	173.2
NMe_2	-365.960014	162.5	-365.785053	162.6	-365.819584	161.8
NO_2 (I) skew	-436.398958	226.6	-436.297916	224.7	-436.331847	223.4
NO_2 (I)	-436.381881	237.4	-436.280477	235.6	-436.313195	235.1
OH	-307.171986	191.0	-307.066568	190.9	-307.096323	190.4
OMe	-346.480737	185.3	-346.347003	185.0	-346.378821	184.4
Ph	-463.001709	184.6	-462.818842	184.4	-462.854251	183.8
SH	-630.149837	188.2	-630.049350	188.4	-630.080127	188.3
SiH_3	-522.614030	207.3	-522.500662	205.6	-522.534515	205.0
SMe	-669.479112	179.2	-669.348962	179.1	-669.381848	178.9
*t*Bu	-389.199567	195.9	-388.986839	194.8	-389.022675	193.8

Table S1b. The total, zero-point vibrational, and Gibbs free energy (hartree) for monosubstituted benzene monocation radicals calculated using the RO-DFT method with the ωB97XD functional and the aug-cc-pVTZ basis set, and relative values referred to energies of the molecules in the ground singlet state calculated at the R-ωB97XD/aug-cc-pVTZ level.

substituent	RO-ωB97XD					
	E	ΔE	ZPE	ΔZPE	G	ΔG
BF_2 (II)	-455.996995	218.2	-455.898990	216.0	-455.933258	215.0
BH_2	-257.340962	212.6	-257.231741	210.7	-257.262606	209.7
$B(OH)_2$	-407.958362	204.4	-407.834648	202.9	-407.869090	201.9
Br	-2805.577367	205.1	-2805.486963	204.6	-2805.518731	204.0
CCH	-308.065559	200.2	-307.955958	199.7	-307.987353	199.0
CF_3 (II)	-568.980984	222.0	-568.877268	220.5	-568.912160	220.3
CFO	-444.456444	247.5	-444.353528	247.1	-444.386000	246.8
CH_3	-271.242883	201.0	-271.116016	199.9	-271.147906	199.7
CHO (II)	-345.223190	219.4	-345.115392	217.6	-345.147835	216.4
Cl	-691.531822	207.1	-691.441067	206.5	-691.471779	205.8
CN	-324.126782	223.0	-324.028813	221.7	-324.060247	221.0
$COCH_3$	-384.561936	211.0	-384.424914	209.8	-384.459058	209.0
$CONH_2$	-400.634243	206.7	-400.506447	206.2	-400.540220	205.3
COOH (II)	-420.491690	217.1	-420.377623	215.4	-420.411077	214.6
COOH (I)	-420.466621	232.8	-420.350902	232.2	-420.383034	232.2
F	-331.161772	209.2	-331.069858	208.5	-331.099687	207.8
H	-231.905844	212.1	-231.807272	210.4	-231.835919	209.2
Li	-238.901444	146.7	-238.811150	146.9	-238.840653	147.0
$MeSO_2$	-819.839760	221.5	-819.702312	220.0	-819.738847	218.9
Na	-393.675182	136.3	-393.585772	136.6	-393.617130	136.5
NC	-324.105941	215.6	-324.008329	214.6	-324.039813	213.8
NH_2	-287.328996	175.6	-287.210332	176.0	-287.240088	175.6
NMe_2	-365.955975	165.1	-365.780907	165.2	-365.815311	164.5
NO_2 (I)	-436.378654	239.4	-436.277733	237.3	-436.310717	236.7
NO_2 (I) skew	-436.395932	228.6	-436.294792	226.6	-436.328774	225.3
OH	-307.168952	192.9	-307.063458	192.8	-307.093211	192.3
OMe	-346.477594	187.3	-346.343744	187.0	-346.375549	186.5
Ph	-462.998450	186.6	-462.815334	186.6	-462.850694	186.0
SH	-630.146979	190.0	-630.046391	190.3	-630.077176	190.2
SiH_3	-522.610983	209.2	-522.497572	207.6	-522.531134	207.1
SMe	-669.476270	181.0	-669.346004	180.9	-669.378909	180.7
*t*Bu	-389.196760	197.7	-388.983866	196.7	-389.019744	195.7

Table S1c. The total, zero-point vibrational, and Gibbs free energy (hartree) for monosubstituted benzenes in the singlet ground state calculated using the R-DFT method with the ⚜B97XD functional and the aug-cc-pVTZ basis set.

substituent	R-ωB97XD		
	E	ZPE	G
BF_2	-456.344703	-456.243244	-456.275839
BH_2	-257.679753	-257.567520	-257.596773
$B(OH)_2$	-408.284015	-408.158021	-408.190862
Br	-2805.904210	-2805.812950	-2805.843790
CCH	-308.384665	-308.274168	-308.304550
CF_3	-569.334799	-569.228599	-569.263305
CFO	-444.850904	-444.747236	-444.779287
CH_3	-271.563272	-271.434530	-271.466103
CHO	-345.572843	-345.462156	-345.492724
Cl	-691.861834	-691.770068	-691.799800
CN	-324.482204	-324.382139	-324.412373
$COCH_3$	-384.898215	-384.759272	-384.792080
$CONH_2$	-400.963645	-400.835037	-400.867375
COOH	-420.837685	-420.720918	-420.752998
F	-331.495167	-331.402052	-331.430833
H	-232.243846	-232.142560	-232.169339
Li	-239.135193	-239.045268	-239.074886
$MeSO_2$	-820.192671	-820.052882	-820.087684
Na	-393.892339	-393.803403	-393.834651
NC	-324.449592	-324.350291	-324.380602
NH_2	-287.608761	-287.490731	-287.519848
NMe_2	-366.219046	-366.044127	-366.077434
NO_2	-436.760144	-436.655963	-436.687880
OH	-307.476422	-307.370738	-307.399679
OMe	-346.776015	-346.641758	-346.672679
Ph	-463.295825	-463.112625	-463.147116
SH	-630.449820	-630.349574	-630.380201
SiH_3	-522.944395	-522.828366	-522.861135
SMe	-669.764644	-669.634322	-669.666880
*t*Bu	-389.511775	-389.297305	-389.331535

Table S1d. The total, zero-point vibrational, and Gibbs free energy (hartree) for monosubstituted benzene monocation radicals in the geometry fixed as in the ground state calculated using the U-DFT method with the ⚜B97XD functional and the aug-cc-pVTZ basis set. The energy differences (kcal/mol) are referred to the appropriate values in the ground state: (doublet (d) *vs*. ground (g) state).

substituent	U-ωB97XD					
	E	$\Delta E_{d\text{-}g}$	ZPE	$\Delta ZPE_{d\text{-}g}$	G	$\Delta G_{d\text{-}g}$
BF_2	-455.992553	220.977	-455.895873	238.432	-455.928874	197.270
BH_2	-257.334140	216.875	-257.226429	232.394	-257.257449	194.572
$B(OH)_2$	-407.953152	207.620	-407.831165	225.713	-407.863874	184.580
Br	-2805.573683	207.408	-2805.483729	225.939	-2805.515497	186.656
CCH	-308.063694	201.412	-307.954813	219.463	-307.987207	180.071
CF_3	-568.976606	224.770	-568.875009	243.659	-568.908712	200.732
CFO	-444.488586	227.358	-444.389576	244.547	-444.423663	203.045
CH_3	-271.239064	203.444	-271.113923	220.996	-271.143819	182.424
CHO	-345.219632	221.643	-345.112079	238.858	-345.143846	199.743
Cl	-691.527199	209.987	-691.437304	227.470	-691.468128	189.470
CN	-324.125084	224.096	-324.028947	240.603	-324.060904	201.578
$COCH_3$	-384.557439	213.840	-384.421306	232.664	-384.453888	191.631
$CONH_2$	-400.622192	214.265	-400.499251	231.001	-400.531355	190.563
COOH	-420.457714	238.435	-420.343556	256.929	-420.376745	215.972
F	-331.156222	212.691	-331.066136	228.851	-331.095911	192.106
H	-231.903152	213.789	-231.806066	227.957	-231.834092	193.567
Li	-238.863036	170.781	-238.775850	187.648	-238.804918	150.822
$MeSO_2$	-819.812278	238.700	-819.676826	257.817	-819.711646	214.129
Na	-393.647661	153.538	-393.560091	172.289	-393.590009	133.907
NC	-324.101349	218.526	-324.005862	235.153	-324.035953	197.250
NH_2	-287.319615	181.442	-287.201352	199.859	-287.231121	162.908
NMe_2	-365.953574	166.586	-365.778532	187.564	-365.810947	146.323
NO_2	-436.358148	252.256	-436.253272	272.721	-436.286062	232.116
OH	-307.163764	196.196	-307.058750	213.936	-307.088528	177.089
OMe	-346.472180	190.659	-346.338382	209.775	-346.370169	170.425
Ph	-462.991981	190.665	-462.809838	211.645	-462.844067	168.523
SH	-630.144328	191.699	-630.044024	210.954	-630.074823	172.409
SiH_3	-522.606262	212.182	-522.494594	230.008	-522.526532	189.404
SMe	-669.474401	182.130	-669.344411	202.352	-669.377363	161.244
*t*Bu	-389.192494	200.352	-388.981551	219.618	-389.015455	176.864

Table S1e. The total, zero-point vibrational, and Gibbs free energy (hartree) for monosubstituted benzene monocation radicals in the geometry fixed as in the ground state calculated using the RO-DFT method with the ⚜B97XD functional and the aug-cc-pVTZ basis set. The energy differences (kcal/mol) are referred to the appropriate values in the ground state: (doublet (d) *vs*. ground (g) state).

substituent	RO-ωB97XD					
	E	$\Delta E_{d\text{-}g}$	ZPE	$\Delta ZPE_{d\text{-}g}$	G	$\Delta G_{d\text{-}g}$
BF_2						
BH_2	-257.330785	218.981	-257.223326	234.342	-257.254479	196.436
$B(OH)_2$	-407.950163	209.495	-407.828188	227.581	-407.860883	186.457
Br	-2805.571143	209.001	-2805.481061	227.613	-2805.512809	188.343
CCH	-308.060177	203.619	-307.951106	221.789	-307.982780	182.849
CF_3	-568.973532	226.698	-568.871874	245.627	-568.905565	202.707
CFO	-444.485521	229.281	-444.386442	246.514	-444.420663	204.928
CH_3	-271.236276	205.193	-271.111043	222.804	-271.140934	184.234
CHO	-345.217032	223.275	-345.109779	240.302	-345.140498	201.843
Cl	-691.524589	211.624	-691.434510	229.223	-691.465283	191.255
CN	-324.121482	226.356	-324.025197	242.957	-324.056947	204.061
$COCH_3$	-384.554957	215.398	-384.418847	234.207	-384.451411	193.186
$CONH_2$	-400.618531	216.562	-400.496503	232.726	-400.529440	191.765
COOH						
F	-331.153442	214.436	-331.063256	230.658	-331.093031	193.914
H	-231.900077	215.718	-231.802924	229.929	-231.830917	195.559
Li	-238.861375	171.823	-238.774194	188.687	-238.803318	151.826
$MeSO_2$	-819.827441	229.185	-819.693392	247.422	-819.727731	204.035
Na	-393.645975	154.596	-393.558419	173.338	-393.588352	134.947
NC	-324.098596	220.253	-324.002676	237.152	-324.033400	198.852
NH_2	-287.316327	183.505	-287.198003	201.961	-287.227770	165.011
NMe_2	-365.949912	168.884	-365.774814	189.897	-365.807218	148.663
NO_2	-436.354680	254.433	-436.250242	274.622	-436.282667	234.247
OH	-307.160842	198.029	-307.055733	215.829	-307.085506	178.986
OMe	-346.469172	192.547	-346.335308	211.704	-346.367107	172.346
Ph	-462.989278	192.361	-462.806920	213.476	-462.841143	170.358
SH	-630.141705	193.345	-630.041306	212.660	-630.072102	174.116
SiH_3	-522.603383	213.988	-522.491628	231.869	-522.523541	191.281
SMe	-669.471837	183.739	-669.341756	204.018	-669.374709	162.910
*t*Bu	-389.189785	202.052	-388.978598	221.471	-389.012473	178.735

Table 1f. The total energy differences (kcal/mol) for monosubstituted benzene mono-cation radicals vs. the ground state energies, the ionization energies without the change of ground state energy and the relaxation energies, calculated using the U- and RO-DFT method with the ⚜B97XD functional and the aug-cc-pVTZ basis set. d stands for doublet and g for ground state.

substituent	U-ωB97XD			RO-ωB97XD		
	ΔE	$\Delta E_{d\text{-}g}$	ΔE_{relax}	ΔE	$\Delta E_{d\text{-}g}$	ΔE_{relax}
BF_2	216.171	220.977	4.806	218.190		
BH_2	210.776	216.875	6.099	212.595	218.981	6.386
$B(OH)_2$	202.374	207.620	5.246	204.350	209.495	5.145
Br	203.393	207.408	4.015	205.096	209.001	3.905
CCH	197.820	201.412	3.592	200.242	203.619	3.377
CF_3	219.976	224.770	4.794	222.022	226.698	4.676
CFO	246.101	227.358	-18.743	247.527	229.281	-18.246
CH_3	199.218	203.444	4.226	201.047	205.193	4.146
CHO	217.464	221.643	4.179	219.410	223.275	3.865
Cl	205.349	209.987	4.638	207.086	211.624	4.538
CN	220.570	224.096	3.526	223.031	226.356	3.325
$COCH_3$	209.014	213.840	4.826	211.019	215.398	4.379
$CONH_2$	204.769	214.265	9.496	206.703	216.562	9.859
COOH	217.120	238.435	21.315	217.115		
F	207.383	212.691	5.308	209.209	214.436	5.227
H	210.053	213.789	3.736	212.099	215.718	3.619
Li	145.624	170.781	25.157	146.680	171.823	25.143
$MeSO_2$	219.431	238.700	19.269	221.455	229.185	7.730
Na	135.207	153.538	18.331	136.268	154.596	18.328
NC	213.798	218.526	4.728	215.644	220.253	4.609
NH_2	173.297	181.442	8.145	175.555	183.505	7.950
NMe_2	162.545	166.586	4.041	165.080		
NO_2	226.648	252.256	25.608	228.937	254.433	25.496
OH	191.037	196.196	5.159	192.940	198.029	5.089
OMe	185.290	190.659	5.369	187.262	192.547	5.285
Ph	184.561	190.665	6.104	186.606	192.361	5.755
SH	188.242	191.699	3.457	190.036	193.345	3.309
SiH_3	207.307	212.182	4.875	209.219	213.988	4.769
SMe	179.174	182.130	2.956	180.957	183.739	2.782
*t*Bu	195.914	200.352	4.438	197.675	202.052	4.377

Table S2a. The CC and C(ipso)-R bond distances (Å) and HOMA aromaticity indices of rings in the radical monocations of monosubstituted benzenes calculated using the U-DFT and RO-DFT approximations the ωB97XD functional and the aug-cc-pVTZ basis set.

	U-ωB97XD/aug-ccpVTZ								R	RO-ωB97XD/aug-ccpVTZ							
R	C1C2	C2C3	C3C4	C4C5	C5C6	C6C1	C_{ipso}-R	HOMA		C1C2	C2C3	C3C4	C4C5	C5C6	C6C1	C_{ipso}-R	HOMA
BF_2 (e)	1.3954	1.4418	1.3739	1.3876	1.4431	1.3806	1.5793	0.782	BF_2 (e)	1.3808	1.4433	1.3782	1.3808	1.4435	1.3853	1.5795	0.770
BH_2 perp	1.4352	1.3595	1.4158	1.4158	1.3595	1.4351	1.5419	0.721	BH_2 perp	1.4346	1.3599	1.4142	1.4142	1.3599	1.4347	1.5412	0.732
$B(OH)_2$	1.4319	1.3595	1.4187	1.4188	1.3595	1.4319	1.6028	0.732	$B(OH)_2$	1.4314	1.3596	1.4173	1.4173	1.3596	1.4314	1.6028	0.741
Br	1.4229	1.3633	1.4095	1.4095	1.3633	1.4229	1.8214	0.831	Br	1.4227	1.3637	1.4078	1.4077	1.3637	1.4227	1.8217	0.838
CCH	1.4303	1.3628	1.4072	1.4072	1.3628	1.4303	1.3817	0.794	CCH	1.4300	1.3635	1.4053	1.4053	1.3635	1.4300	1.3833	0.803
CF_3 (II)	1.4263	1.4179	1.3615	1.4210	1.4234	1.3571	1.5202	0.766	CF_3 (II)	1.4255	1.4167	1.3616	1.4203	1.4221	1.3573	1.5202	0.775
CF_3 (e) av	1.3917	1.4207	1.3913	1.3913	1.4207	1.3917	1.5202	0.945		1.3914	1.4194	1.3910	1.3910	1.4194	1.3914	1.5202	0.950
CFO	1.4109	1.3743	1.3929	1.3950	1.3720	1.4114	1.4042	0.934	CFO	1.4109	1.3743	1.3929	1.3950	1.3720	1.4114	1.4042	0.934
CH_3	1.4317	1.3589	1.4161	1.4161	1.3589	1.4317	1.4701	0.739	CH_3	1.4312	1.3591	1.4148	1.4148	1.3591	1.4312	1.4695	0.747
CHO (I) LOC	1.4083	1.3756	1.3914	1.3966	1.3717	1.4113	1.4170	0.939	CHO (II)	1.3639	1.4067	1.4300	1.3650	1.4042	1.4385	1.5008	0.780
CHO (I) MIN skew	1.3923	1.3730	1.4335	1.3857	1.3750	1.4414	1.4898	0.805	CHO (e) av	1.4012	1.40545	1.3975	1.3975	1.4055	1.4012	1.5008	0.991
Cl	1.4251	1.3610	1.4130	1.4130	1.3610	1.4251	1.6708	0.798	Cl	1.4248	1.3613	1.4113	1.4113	1.3613	1.4248	1.6707	0.806
CN	1.4305	1.3597	1.4156	1.4156	1.3597	1.4305	1.4013	0.752	CN	1.4300	1.3599	1.4145	1.4145	1.3599	1.4301	1.4048	0.760
$COCH_3$ (I) LOC	1.4019	1.3777	1.3907	1.3921	1.3752	1.4065	1.4393	0.958									
$COCH_3$ (I) MIN skew	1.4055	1.3686	1.4126	1.3901	1.3750	1.4193	1.4949	0.908	$COCH_3$ (I)	1.4056	1.3690	1.4104	1.3891	1.3760	1.4164	1.4982	0.918
$CONH_2$ skew	1.4098	1.3687	1.4066	1.3957	1.3721	1.4151	1.4827	0.915	$CONH_2$ skew	1.4098	1.3693	1.3947	1.4046	1.3726	1.4138	1.4841	0.921
COOH (II) MIN	1.3612	1.4225	1.4202	1.3617	1.4194	1.4251	1.5032	0.783	COOH (II) MIN	1.4241	1.4183	1.3620	1.4190	1.4214	1.3615	1.5031	0.794
COOH (e) av	1.3932	1.4210	1.3910	1.3910	1.4210	1.3932	1.5032	0.944	COOH (e) av	1.3928	1.4199	1.3905	1.3905	1.4199	1.3928	1.5031	0.948
COOH (I) LOC	1.4041	1.3771	1.3913	1.3923	1.3752	1.4050	1.4253	0.957	COOH (I) LOC	1.4041	1.3770	1.3914	1.3923	1.3752	1.4050	1.4251	0.957
F	1.4207	1.3588	1.4196	1.4196	1.3588	1.4207	1.2873	0.781	F	1.4202	1.3591	1.4184	1.4183	1.3589	1.4201	1.2871	0.790
H	1.4226	1.3597	1.4225	1.4224	1.3597	1.4226	1.0825	0.767	H	1.4214	1.3599	1.4215	1.4214	1.3599	1.4215	1.0825	0.778
H (e) imag	1.3802	1.4443	1.3802	1.3802	1.4443	1.3802	1.0795	0.758	H (e) imag	1.3793	1.4445	1.3793	1.3793	1.4445	1.3793	1.0795	0.751
Li ovp	1.3745	1.4023	1.3956	1.3957	1.4023	1.3745	2.3772	0.956	Li ovp	1.3740	1.4022	1.3955	1.3955	1.4022	1.3740	2.3751	0.954
$MeSO_2$ skew	1.4144	1.3641	1.4119	1.4057	1.3659	1.4192	1.7803	0.865	$MeSO_2$	1.4148	1.3644	1.4105	1.4045	1.3662	1.4194	1.7826	0.868
Na ovp	1.3729	1.4001	1.3934	1.3934	1.4001	1.3729	2.8217	0.951	Na ovp	1.3729	1.4001	1.3934	1.3934	1.4001	1.3729	2.8217	0.951
NC	1.4295	1.3590	1.4156	1.4156	1.3590	1.4295	1.3372	0.754	NC	1.4291	1.3591	1.4141	1.4141	1.3591	1.4291	1.3376	0.761
NH_2	1.4294	1.3622	1.4065	1.4065	1.3622	1.4294	1.3240	0.797	NH_2	1.4291	1.3619	1.4049	1.4049	1.3619	1.4291	1.3242	0.800
NMe_2	1.4257	1.3678	1.3984	1.3984	1.3678	1.4257	1.3438	0.856	NMe_2	1.4242	1.3683	1.3963	1.3963	1.3683	1.4242	1.3452	0.866
NO_2 (I) skew MIN	1.4159	1.3608	1.4184	1.4188	1.3608	1.4155	1.4512	0.817	NO_2 (I) skew MIN	1.4152	1.3613	1.4168	1.4168	1.3613	1.4152	1.4507	0.828
NO_2 (I) LOC	1.4046	1.3725	1.3963	1.3963	1.3725	1.4050	1.3754	0.945	NO_2 (I)	1.4045	1.3588	1.3962	1.3962	1.3726	1.4044	1.3753	0.910
OH	1.4267	1.3588	1.4161	1.4098	1.3603	1.4296	1.2993	0.772	OH	1.4262	1.3588	1.4150	1.4083	1.3600	1.4298	1.2995	0.775

OMe	1.4260	1.3610	1.4144	1.4051	1.3614	1.4340	1.2924	0.778	OMe	1.4258	1.3610	1.4133	1.4036	1.3609	1.4341	1.2926	0.779
Ph	1.4246	1.3682	1.3983	1.3983	1.3682	1.4247	1.4304	0.863	Ph	1.4238	1.3689	1.3969	1.3969	1.3689	1.4238	1.4324	0.871
SH	1.4214	1.3655	1.4046	1.4018	1.3666	1.4241	1.6965	0.857	SH	1.4209	1.3659	1.3999	1.4029	1.3670	1.4236	1.6976	0.863
SiH_3	1.4270	1.3607	1.4225	1.4122	1.3603	1.4398	1.9075	0.728	SiH_3	1.4263	1.3609	1.4213	1.4107	1.3605	1.4394	1.9070	0.737
SMe	1.4145	1.3689	1.4021	1.3961	1.3703	1.4218	1.6968	0.895	SMe	1.4134	1.3697	1.4003	1.3941	1.3711	1.4205	1.6990	0.904
*t*Bu	1.4263	1.3602	1.4185	1.4070	1.3599	1.4424	1.4925	0.731	*t*Bu	1.4257	1.3605	1.4172	1.4056	1.3599	1.4419	1.4919	0.739

Table S2b. Juxtaposition of the HOMA and NICS$_{zz}$ (ppm) aromaticity parameters for the monosubstituted benzenes in the ground and cation radical states calculated at the R-⚜B97XD/aug-cc-pVTZ and U-⚜B97XD/aug-cc-pVTZ levels.

Ground state				Cation radicals							
								Order according to increasing NICS$_{zz}$(1)			
Substituent	HOMA [59]	NICS$_{zz}$(0)	NICS$_{zz}$(1)	Substituent	HOMA	NICS$_{zz}$(0)	NICS$_{zz}$(1)	Substituent	HOMA	NICS$_{zz}$(0)	NICS$_{zz}$(1)
BF_2	0.991	-14.0	-29.2	BF_2 (II)	0.782	60.7	35.1	Na	0.951	-20.5	-35.2
BH_2	0.980	-14.1	-29.0	BH_2	0.721	42.2	19.5	Li	0.956	-19.6	-33.6
$B(OH)_2$	0.993	-13.9	-29.2	$B(OH)_2$	0.732	66.1	39.9	COOH (I) LOC	0.957	-9.3	-24.8
Br	1.000	-14.9	-28.2	Br	0.831	25.3	5.5	$COCH_3$ (I) LOC	0.958	-9.9	-24.7
CCH	0.994	-14.4	-28.5	CCH	0.794	19.4	0.0	CHO (I) LOC	0.939	-8.9	-23.7
CF_3	0.999	-15.1	-29.6	CF_3 (II)	0.766	58.9	33.9	CFO	0.934	-7.2	-23.2
CFO	0.997	-13.9	-28.8	CFO	0.934	-7.2	-23.2	NO_2 (I) LOC	0.945	-3.9	-20.3
CH_3	0.998	-14.9	-29.0	CH_3	0.739	41.8	19.2	SMe	0.895	6.0	-12.0
CHO	0.996	-13.4	-28.7	CHO (I) LOC	0.939	-8.9	-23.7	NMe_2	0.856	6.5	-11.3
				CHO (I) MINskew	0.805	49.8	24.8	Ph	0.863	8.6	-10.0
Cl	0.999	-15.2	-28.4	Cl	0.798	30.1	9.8	SH	0.857	10.7	-7.4
CN	0.996	-14.9	-29.0	CN	0.752	40.1	18.0	NH_2	0.797	13.0	-5.5
$COCH_3$	0.996	-13.5	-28.6	$COCH_3$ (I) LOC	0.958	-9.9	-24.7	$CONH_2$	0.915	10.6	-5.4
				$COCH_3$ (I) MIN	0.908	15.4	-4.5	$COCH_3$(I)MINskew	0.908	15.4	-4.5
$CONH_2$	0.998	-14.4	-28.9	$CONH_2$	0.915	10.6	-5.4	CCH	0.794	19.4	0.0
COOH	0.998	-13.9	-28.9	COOH (I) LOC	0.957	-9.3	-24.8	OMe	0.778	20.8	1.7
				COOH (II) MIN	0.783	59.3	34.1	OH	0.772	22.9	3.7
F	0.997	-16.5	-29.2	F	0.781	35.7	15.3	Br	0.831	25.3	5.5
H	1.000	-16.2	-30.5	H	0.767	73.2	46.4	$MeSO_2$ skew	0.865	27.7	8.7
				H (e) imag	0.758	65.3	39.5	Cl	0.798	30.1	9.8
Li	0.968	-15.9	-29.6	Li	0.956	-19.6	-33.6	NC	0.754	33.7	13.1
$MeSO_2$	1.000	-15.4	-29.1	$MeSO_2$	0.865	27.7	8.7	F	0.781	35.7	15.3
Na	0.972	-16.2	-29.4	Na	0.951	-20.5	-35.2	CN	0.752	40.1	18.0
NC	0.999	-15.6	-28.8	NC	0.754	33.7	13.1	CH_3	0.739	41.8	19.2
NH_2	0.994	-11.6	-26.3	NH_2	0.797	13.0	-5.5	BH_2 perp	0.721	42.2	19.5
NMe_2	0.974	-12.2	-27.5	NMe_2	0.856	6.5	-11.3	*t*Bu	0.731	42.4	19.6
NO_2	0.998	-14.9	-28.9	NO_2 (I) LOC	0.945	-3.9	-20.3	NO_2 (I) skew MIN	0.817	43.5	22.0
				NO_2 (I) skew MIN	0.817	43.5	22.0	CHO (I) MIN skew	0.805	49.8	24.8
OH	0.999	-14.2	-27.7	OH	0.772	22.9	3.7	SiH_3	0.728	56.5	31.8
OMe	0.993	-14.3	-28.2	OMe	0.778	20.8	1.7	CF_3 (II)	0.766	58.9	33.9
Ph	0.995	-14.0	-28.5	Ph	0.863	8.6	-10.0	COOH (II) MIN	0.783	59.3	34.1
SH	0.999	-13.2	-27.3	SH	0.857	10.7	-7.4	BF_2 (e)	0.782	60.7	35.1
SiH_3	0.993	-14.6	-29.4	SiH_3	0.728	56.5	31.8	H (e) imag	0.758	65.3	39.5
SMe	0.995	-12.5	-27.5	SMe	0.895	6.0	-12.0	$B(OH)_2$	0.732	66.1	39.9
*t*Bu	0.994	-14.0	-28.9	*t*Bu	0.731	42.4	19.6	H	0.767	73.2	46.4

Table S3a. The NBO partial charges in the radical monocations of monosubstituted benzenes calculated using the U-DFT approximation, the ωB97XD functional and the aug-cc-pVTZ basis set.

subst	C1	C2	C6	C3	C5	C4	H2	H6	H3	H5	H4	X1	X2	X3	X3	X4	X5	X6	X7	X8
BF_2	-0.460	0.017	0.071	0.026	-0.031	-0.237	0.248	0.245	0.243	0.247	0.260	1.242	-0.435	-0.436						
BH_2	-0.113	-0.147	-0.147	-0.172	-0.172	0.054	0.242	0.242	0.252	0.252	0.237	0.470	0.002	0.002						
$B(OH)_2$	-0.030	-0.140	-0.139	-0.183	-0.183	0.079	0.254	0.254	0.249	0.249	0.233	1.023	-0.845	-0.845	0.511	0.511				
Br	-0.025	-0.167	-0.167	-0.178	-0.178	0.021	0.256	0.256	0.253	0.253	0.240	0.434								
CCH	-0.004	-0.088	-0.088	-0.198	-0.198	0.018	0.248	0.248	0.251	0.251	0.238	-0.020	0.075	0.268						
CF_3(II)	-0.095	0.102	-0.153	-0.162	0.093	-0.153	0.249	0.269	0.258	0.241	0.257	1.008	-0.306	-0.306	-0.302					
CFO	-0.260	-0.087	-0.077	-0.202	-0.199	-0.052	0.245	0.240	0.249	0.248	0.241	0.855	-0.001	-0.200						
CH_3	0.252	-0.158	-0.158	-0.174	-0.174	0.052	0.244	0.244	0.251	0.251	0.236	-0.668	0.256	0.256	0.291					
CHO (I) LOC	-0.208	-0.080	-0.091	-0.206	-0.195	-0.056	0.234	0.240	0.248	0.248	0.240	0.362	-0.027	0.293						
CHO (I) MIN skew	0.040	-0.004	-0.217	-0.243	-0.051	0.014	0.259	0.254	0.259	0.247	0.243	0.384	-0.355	0.171						
Cl	0.083	-0.160	-0.160	-0.175	-0.175	0.037	0.259	0.259	0.255	0.255	0.240	0.280								
CN	0.048	-0.102	-0.102	-0.179	-0.179	0.071	0.261	0.261	0.259	0.259	0.242	0.212	-0.053							
$COCH_3$ (I) LOC	-0.200	-0.111	-0.102	-0.195	-0.193	-0.081	0.229	0.235	0.242	0.244	0.238	0.566	-0.069	-0.619	0.275	0.275	0.266			
$COCH_3$ (I) MIN	0.007	-0.184	-0.087	-0.139	-0.214	-0.029	0.243	0.249	0.247	0.251	0.240	0.588	-0.278	-0.691	0.266	0.260	0.270			
$CONH_2$	0.007	-0.164	-0.112	-0.168	-0.203	-0.033	0.245	0.247	0.245	0.248	0.238	0.629	-0.327	-0.717	0.434	0.428				
COOH (II) Min	-0.123	-0.137	0.115	0.081	-0.161	-0.158	0.267	0.259	0.239	0.256	0.255	0.766	-0.523	-0.654	0.519					
COOH (I) LOC	-0.226	-0.100	-0.105	-0.196	-0.196	-0.079	0.242	0.235	0.243	0.244	0.238	0.764	-0.049	-0.549	0.533					
F	0.610	-0.207	-0.207	-0.161	-0.161	0.051	0.268	0.268	0.257	0.257	0.241	-0.216								
H	0.087	-0.166	-0.166	-0.166	-0.166	0.087	0.237	0.254	0.254	0.254	0.254	0.237								
H (e)	-0.255	0.005	0.005	0.005	0.005	-0.255	0.243	0.243	0.243	0.243	0.259	0.259								
Li	0.175	-0.347	-0.347	-0.217	-0.217	-0.253	0.265	0.265	0.252	0.252	0.253	0.919								
$MeSO_2$	-0.103	-0.171	-0.140	-0.168	-0.183	0.022	0.254	0.269	0.247	0.254	0.240	2.112	-0.864	-0.748	-0.828	0.254	0.287	0.267		
Na	0.167	-0.349	-0.349	-0.212	-0.213	-0.248	0.254	0.254	0.240	0.240	0.241	0.975								
NC	0.313	-0.130	-0.130	-0.174	-0.174	0.055	0.262	0.262	0.258	0.258	0.241	-0.573	0.533							
NH2	0.243	-0.165	-0.165	-0.179	-0.179	-0.035	0.236	0.236	0.247	0.247	0.237	-0.568	0.422	0.422						
NMe_2	0.192	-0.168	-0.168	-0.183	-0.183	-0.071	0.232	0.232	0.241	0.241	0.234	-0.196	-0.404	-0.404	0.238	0.234	0.231	0.238	0.234	0.231
NO_2 (I) LOC	0.005	-0.105	-0.105	-0.192	-0.192	-0.041	0.257	0.257	0.253	0.253	0.244	0.484	-0.059	-0.059						
NO_2 (I) MIN skew	0.281	-0.162	-0.163	-0.159	-0.158	0.070	0.272	0.272	0.260	0.260	0.243	0.484	-0.250	-0.250						
OH	0.461	-0.214	-0.160	-0.159	-0.184	0.009	0.244	0.259	0.252	0.253	0.239	-0.513	0.513							
OMe	0.449	-0.140	-0.229	-0.198	-0.155	-0.008	0.253	0.246	0.251	0.247	0.237	-0.354	-0.247	0.230	0.209	0.209				
Ph	0.014	-0.121	-0.121	-0.196	-0.196	-0.042	0.225	0.225	0.240	0.240	0.233	0.014	0.487							
SH	-0.155	-0.146	-0.156	-0.187	-0.179	-0.023	0.245	0.238	0.250	0.249	0.239	0.464	0.159							
SiH_3	-0.151	-0.184	-0.135	-0.151	-0.198	0.070	0.244	0.240	0.249	0.252	0.235	0.867	-0.125	-0.105	-0.108					
SMe	-0.206	-0.133	-0.173	-0.195	-0.173	-0.051	0.241	0.233	0.246	0.243	0.237	0.712	-0.757	0.266	0.255	0.255				
tBu	0.280	-0.198	-0.133	-0.146	-0.205	0.041	0.249	0.239	0.246	0.249	0.234	-0.124	0.268							

Table S3b. The NBO partial charges in the radical monocations of monosubstituted benzenes calculated using the U-DFT approximation, the ωB97XD functional and the aug-cc-pVTZ basis set. C(ipso), C(o), C(m), C(p), C(2o+2m), C(ip+p), Σ(C), Σ(H), X1 and R denote partial charge at C_{ipso} atom, averaged partial charge over C_{ortho} atoms, over C_{meta} atoms, sum of partial charges of all ortho and meta atoms, sum for the ipso and para atoms, sum of all carbon ring atoms, all ring H atoms, the atom attached in the ipso position and sum of partial charges of all substituent atoms, respectively.

subst	C(ipso)	C(o)	C(m)	C(p)	C(2o+2m)	C(ip+p)	Σ(C)	Σ(H)	X1	R
BF_2	-0.460	0.044	-0.003	-0.237	0.083	-0.697	**-0.614**	1.243	1.242	0.371
BH_2	-0.113	-0.147	-0.172	0.054	-0.638	-0.059	**-0.697**	1.225	0.470	0.474
$B(OH)_2$	-0.030	-0.140	-0.183	0.079	-0.645	0.049	**-0.596**	1.239	1.023	0.355
Br	-0.025	-0.167	-0.178	0.021	-0.690	-0.004	**-0.694**	1.258	0.434	0.434
CCH	-0.004	-0.088	-0.198	0.018	-0.572	0.014	**-0.558**	1.236	-0.020	0.323
CF_3(II)	-0.095	-0.026	-0.035	-0.153	-0.120	-0.248	**-0.368**	1.274	1.008	0.094
CFO	-0.260	-0.082	-0.201	-0.052	-0.565	-0.312	**-0.877**	1.223	0.855	0.654
CH_3	0.252	-0.158	-0.174	0.052	-0.664	0.304	**-0.360**	1.226	-0.668	0.135
CHO (I) LOC	-0.208	-0.086	-0.201	-0.056	-0.572	-0.264	**-0.836**	1.210	0.362	0.628
CHO (I) MIN skew	0.040	-0.111	-0.147	0.014	-0.515	0.054	**-0.461**	1.262	0.384	0.200
Cl	0.083	-0.160	-0.175	0.037	-0.670	0.120	**-0.550**	1.268	0.280	0.280
CN	0.048	-0.102	-0.179	0.071	-0.562	0.119	**-0.443**	1.282	0.212	0.159
$COCH_3$ (I) LOC	-0.200	-0.107	-0.194	-0.081	-0.601	-0.281	**-0.882**	1.188	0.566	0.694
$COCH_3$ (I) MIN	0.007	-0.136	-0.177	-0.029	-0.624	-0.022	**-0.646**	1.230	0.588	0.415
$CONH_2$	0.007	-0.138	-0.186	-0.033	-0.647	-0.026	**-0.673**	1.223	0.629	0.447
COOH (II) Min	-0.123	-0.011	-0.040	-0.158	-0.102	-0.281	**-0.383**	1.276	0.766	0.108
COOH (I) LOC	-0.226	-0.103	-0.196	-0.079	-0.597	-0.305	**-0.902**	1.202	0.764	0.699
F	0.610	-0.207	-0.161	0.051	-0.736	0.661	**-0.075**	1.291	-0.216	-0.216
H	0.087	-0.166	-0.166	0.087	-0.664	0.174	**-0.490**	1.253	0.237	0.237
H (e)	-0.255	0.005	0.005	-0.255	0.020	-0.510	**-0.490**	1.231	0.259	0.259
Li	0.175	-0.347	-0.217	-0.253	-1.128	-0.078	**-1.206**	1.287	0.919	0.919
$MeSO_2$	-0.103	-0.156	-0.176	0.022	-0.662	-0.081	**-0.743**	1.264	2.112	0.480
Na	0.167	-0.349	-0.213	-0.248	-1.123	-0.081	**-1.204**	1.229	0.975	0.975
NC	0.313	-0.130	-0.174	0.055	-0.608	0.368	**-0.240**	1.281	-0.573	-0.040
NH2	0.243	-0.165	-0.179	-0.035	-0.688	0.208	**-0.480**	1.203	-0.568	0.276
NMe_2	0.192	-0.168	-0.183	-0.071	-0.702	0.121	**-0.581**	1.180	-0.196	0.402
NO_2 (I) LOC	0.005	-0.105	-0.192	-0.041	-0.594	-0.036	**-0.630**	1.264	0.484	0.366
NO_2 (I) MIN skew	0.281	-0.163	-0.159	0.070	-0.642	0.351	**-0.291**	1.307	0.484	-0.016
OH	0.461	-0.187	-0.172	0.009	-0.717	0.470	**-0.247**	1.247	-0.513	0.000

OMe	0.449	-0.185	-0.177	-0.008	-0.722	0.441	**-0.281**	1.234	-0.354	0.047
Ph	0.014	-0.121	-0.196	-0.042	-0.634	-0.028	**-0.662**	1.163	0.014	0.501
SH	-0.155	-0.151	-0.183	-0.023	-0.668	-0.178	**-0.846**	1.221	0.464	0.623
SiH_3	-0.151	-0.160	-0.175	0.070	-0.668	-0.081	**-0.749**	1.220	0.867	0.529
SMe	-0.206	-0.153	-0.184	-0.051	-0.674	-0.257	**-0.931**	1.200	0.712	0.731
tBu	0.280	-0.166	-0.176	0.041	-0.682	0.321	**-0.361**	1.217	-0.124	0.144

Table S3c. The NBO partial charges in the radical monocations of monosubstituted benzenes calculated using the RO-DFT approximation, the ωB97XD functional and the aug-cc-pVTZ basis set.

subst	C1	C2	C6	C3	C5	C4	H2	H6	H3	H5	H4	X1	X2	X3	X3	X4	X5	X6	X7	X8
BF_2 (e) MIN	-0.462	0.050	0.039	-0.009	0.003	-0.238	0.247	0.247	0.245	0.245	0.260	1.243	-0.435	-0.435						
BH_2	-0.115	-0.145	-0.146	-0.176	-0.176	0.057	0.242	0.242	0.252	0.252	0.237	0.470	0.003	0.003						
$B(OH)_2$	-0.027	-0.139	-0.139	-0.187	-0.187	0.081	0.254	0.254	0.249	0.249	0.233	1.023	-0.844	-0.844	0.511	0.511				
Br	-0.026	-0.162	-0.162	-0.183	-0.183	0.023	0.256	0.256	0.254	0.254	0.241	0.434								
CCH	-0.004	-0.084	-0.084	-0.203	-0.203	0.019	0.248	0.248	0.251	0.251	0.238	-0.022	0.077	0.269						
CF_3 (II)	-0.095	0.105	-0.156	-0.166	0.096	-0.153	0.250	0.269	0.259	0.242	0.257	1.008	-0.306	-0.302	-0.306					
CFO	-0.260	-0.077	-0.087	-0.203	-0.199	-0.051	0.245	0.240	0.249	0.248	0.241	0.856	-0.003	-0.201						
CH_3	0.251	-0.158	-0.158	-0.177	-0.177	0.056	0.244	0.244	0.251	0.251	0.237	-0.667	0.256	0.292	0.256					
CHO	-0.066	-0.192	0.107	0.072	-0.204	-0.105	0.255	0.257	0.241	0.259	0.253	0.399	-0.424	0.150						
Cl	0.082	-0.157	-0.157	-0.180	-0.180	0.040	0.259	0.259	0.255	0.255	0.241	0.280								
CN	0.060	-0.104	-0.104	-0.182	-0.182	0.074	0.262	0.262	0.260	0.260	0.243	0.206	-0.055							
$COCH_3$	0.004	-0.179	-0.091	-0.149	-0.212	-0.034	0.243	0.249	0.247	0.250	0.240	0.597	-0.276	-0.691	0.266	0.262	0.274			
$CONH_2$	0.004	-0.156	-0.112	-0.175	-0.203	-0.034	0.245	0.247	0.247	0.248	0.239	0.632	-0.328	-0.716	0.435	0.428				
COOH (II) MIN	-0.124	-0.140	0.117	0.084	-0.163	-0.160	0.267	0.260	0.239	0.256	0.255	0.766	-0.523	-0.654	0.519					
COOH (I) LOC	-0.192	0.068	0.017	-0.058	0.033	-0.209	0.260	0.276	0.250	0.248	0.256	0.738	-0.574	-0.647	0.535					
F	0.610	-0.208	-0.208	-0.164	-0.164	0.056	0.268	0.268	0.257	0.257	0.242	-0.215								
H	0.090	-0.168	-0.168	-0.168	-0.168	0.090	0.254	0.254	0.254	0.254	0.237	0.237								
H (e) imag	-0.253	0.004	0.004	0.004	0.004	-0.253	0.243	0.243	0.243	0.243	0.259	0.259								
Li	0.174	-0.347	-0.347	-0.217	-0.217	-0.253	0.266	0.266	0.252	0.252	0.253	0.919								
$MeSO_2$	-0.098	-0.166	-0.137	-0.174	-0.186	0.026	0.247	0.270	0.252	0.255	0.241	2.115	-0.865	-0.759	-0.828	0.254	0.267	0.287		
Na	0.167	-0.349	-0.349	-0.212	-0.213	-0.248	0.254	0.254	0.240	0.240	0.241	0.975								
NC	0.313	-0.128	-0.128	-0.179	-0.179	0.057	0.262	0.262	0.258	0.258	0.242	-0.574	0.536							
NH2	0.244	-0.161	-0.161	-0.183	-0.183	-0.027	0.237	0.237	0.248	0.248	0.238	-0.579	0.422	0.422						
NMe_2	0.188	-0.162	-0.162	-0.187	-0.187	-0.067	0.233	0.233	0.241	0.241	0.235	-0.204	-0.403	0.230	0.237	0.234	-0.403	0.230	0.237	0.234
NO_2 (I) MIN	0.040	-0.007	-0.007	-0.003	-0.003	-0.215	0.267	0.267	0.255	0.255	0.258	0.495	-0.301	-0.301						
NO_2 (I) LOC	-0.010	0.005	0.005	-0.030	-0.029	-0.184	0.280	0.280	0.256	0.256	0.258	0.450	-0.268	-0.269						
OH	0.461	-0.213	-0.158	-0.163	-0.187	0.016	0.244	0.259	0.252	0.254	0.240	-0.517	0.513							
OMe	0.450	-0.137	-0.227	-0.202	-0.160	-0.001	0.253	0.245	0.251	0.248	0.238	-0.360	-0.245	0.209	0.230	0.209				
Ph	0.011	-0.118	-0.118	-0.199	-0.199	-0.041	0.225	0.225	0.240	0.240	0.233	0.011	0.488							
SH	-0.158	-0.150	-0.139	-0.184	-0.190	-0.020	0.238	0.245	0.250	0.250	0.240	0.459	0.159							
SiH_3	-0.151	-0.183	-0.135	-0.155	-0.201	0.072	0.244	0.240	0.250	0.252	0.236	0.867	-0.124	-0.107	-0.105					
SMe	-0.211	-0.167	-0.128	-0.178	-0.198	-0.050	0.233	0.241	0.244	0.246	0.238	0.710	-0.757	0.255	0.255	0.266				
*t*Bu	0.277	-0.196	-0.133	-0.152	-0.207	0.044	0.248	0.239	0.246	0.249	0.235	-0.123	0.273							

Table S3d. The NBO partial charges in the radical monocations of monosubstituted benzenes calculated using the RO-DFT approximation, the ωB97XD functional and the aug-cc-pVTZ basis set. C(ipso), C(o), C(m), C(p), C(2o+2m), C(ip+p), Σ(C), Σ(H), X1 and R denote partial charge at C_{ipso} atom, averaged partial charge over C_{ortho} atoms, over C_{meta} atoms, sum of partial charges of all ortho and meta atoms, sum for the ipso and para atoms, sum of all carbon ring atoms, all ring H atoms, the atom attached in the ipso position and sum of partial charges of all substituent atoms, respectively.

subst	C(ipso)	C(o)	C(m)	C(p)	C(2o+2m)	C(ip+p)	Σ(C)	Σ(H)	X1	R
BF_2 (e) MIN	-0.462	0.045	-0.003	-0.238	0.083	-0.700	**-0.617**	1.244	1.243	0.373
BH_2	-0.115	-0.146	-0.176	0.057	-0.643	-0.058	**-0.701**	1.225	0.470	0.476
$B(OH)_2$	-0.027	-0.139	-0.187	0.081	-0.652	0.054	**-0.598**	1.239	1.023	0.357
Br	-0.026	-0.162	-0.183	0.023	-0.690	-0.003	**-0.693**	1.261	0.434	0.434
CCH	-0.004	-0.084	-0.203	0.019	-0.574	0.015	**-0.559**	1.236	-0.022	0.324
CF_3 (II)	-0.095	-0.026	-0.035	-0.153	-0.121	-0.248	**-0.369**	1.277	1.008	0.094
CFO	-0.260	-0.082	-0.201	-0.051	-0.566	-0.311	**-0.877**	1.223	0.856	0.652
CH_3	0.251	-0.158	-0.177	0.056	-0.670	0.307	**-0.363**	1.227	-0.667	0.137
CHO	-0.066	-0.043	-0.066	-0.105	-0.217	-0.171	**-0.388**	1.265	0.399	0.125
Cl	0.082	-0.157	-0.180	0.040	-0.674	0.122	**-0.552**	1.269	0.280	0.280
CN	0.060	-0.104	-0.182	0.074	-0.572	0.134	**-0.438**	1.287	0.206	0.151
$COCH_3$	0.004	-0.135	-0.181	-0.034	-0.631	-0.030	**-0.661**	1.229	0.597	0.432
$CONH_2$	0.004	-0.134	-0.189	-0.034	-0.646	-0.030	**-0.676**	1.226	0.632	0.451
COOH (II) MIN	-0.124	-0.012	-0.040	-0.160	-0.102	-0.284	**-0.386**	1.277	0.766	0.108
COOH (I) LOC	-0.192	0.043	-0.013	-0.209	0.060	-0.401	**-0.341**	1.290	0.738	0.052
F	0.610	-0.208	-0.164	0.056	-0.744	0.666	**-0.078**	1.292	-0.215	-0.215
H	0.090	-0.168	-0.168	0.090	-0.672	0.180	**-0.492**	1.253	0.237	0.237
H (e) imag	-0.253	0.004	0.004	-0.253	0.016	-0.506	**-0.490**	1.231	0.259	0.259
Li	0.174	-0.347	-0.217	-0.253	-1.128	-0.079	**-1.207**	1.289	0.919	0.919
$MeSO_2$	-0.098	-0.152	-0.180	0.026	-0.663	-0.072	**-0.735**	1.265	2.115	0.471
Na	0.167	-0.349	-0.213	-0.248	-1.123	-0.081	**-1.204**	1.229	0.975	0.975
NC	0.313	-0.128	-0.179	0.057	-0.614	0.370	**-0.244**	1.282	-0.574	-0.038
NH2	0.244	-0.161	-0.183	-0.027	-0.688	0.217	**-0.471**	1.208	-0.579	0.265
NMe_2	0.188	-0.162	-0.187	-0.067	-0.698	0.121	**-0.577**	1.183	-0.204	0.392
NO_2 (I) MIN	0.040	-0.007	-0.003	0.267	-0.020	0.307	**0.287**	1.302	0.495	-0.107
NO_2 (I) LOC	-0.010	0.005	-0.030	-0.184	-0.049	-0.194	**-0.243**	1.330	0.450	-0.087
OH	0.461	-0.186	-0.175	0.016	-0.721	0.477	**-0.244**	1.249	-0.517	-0.004
OMe	0.450	-0.182	-0.181	-0.001	-0.726	0.449	**-0.277**	1.235	-0.360	0.043
Ph	0.011	-0.118	-0.199	-0.041	-0.634	-0.030	**-0.664**	1.163	0.011	0.499
SH	-0.158	-0.145	-0.187	-0.020	-0.663	-0.178	**-0.841**	1.223	0.459	0.618
SiH_3	-0.151	-0.159	-0.178	0.072	-0.674	-0.079	**-0.753**	1.222	0.867	0.531

SMe	-0.211	-0.148	-0.188	-0.050	-0.671	-0.261	**-0.932**	1.202	0.710	0.729
*t*Bu	0.277	-0.165	-0.180	0.044	-0.688	0.321	**-0.367**	1.217	-0.123	0.150

Table S4a. The NBO partial spins in the radical monocations of monosubstituted benzenes calculated using the U-DFT approximation, the ωB97XD functional and the aug-cc-pVTZ basis set.

subst	C1	C2	C6	C3	C5	C4	H2	H6	H3	H5	H4	X1	X2	X3	X3	X4	X5	X6	X7	X8
BF_2	-0.101	0.358	0.267	0.362	0.261	-0.107	-0.008	-0.011	-0.008	-0.011	0.002	-0.001	-0.002	-0.001						
BH_2	0.374	0.082	0.081	-0.014	-0.014	0.437	-0.003	-0.003	0.000	0.000	-0.013	-0.010	0.042	0.042						
$B(OH)_2$	0.458	0.039	0.039	0.014	0.015	0.436	-0.002	-0.002	0.001	-0.001	-0.013	-0.017	0.019	0.019	-0.001	-0.001				
Br	0.243	0.092	0.092	-0.036	-0.036	0.367	-0.003	-0.003	0.001	0.001	-0.011	0.294								
CCH	0.241	0.090	0.090	-0.040	-0.040	0.322	-0.003	-0.003	0.001	0.001	-0.010	-0.057	0.423	-0.015						
CF_3(II)	0.033	0.451	0.030	0.021	0.456	0.031	-0.013	-0.001	-0.001	-0.014	-0.002	-0.002	0.004	0.004	-0.001					
CFO	0.029	-0.001	-0.008	0.008	0.005	-0.014	0.000	0.000	0.000	0.001	0.000	-0.043	0.976	0.047						
CH_3	0.378	0.085	0.085	-0.017	-0.017	0.443	-0.003	-0.003	0.000	0.000	-0.013	0.004	0.008	0.008	0.042					
CHO (I) LOC	0.073	-0.008	-0.012	0.008	0.010	-0.021	0.001	0.000	0.001	0.001	0.001	-0.044	0.878	0.113						
CHO (I) MIN skew	0.324	0.233	-0.101	-0.092	0.225	0.280	-0.007	0.002	0.002	-0.006	-0.008	-0.077	0.219	0.005						
Cl	0.285	0.089	0.089	-0.029	-0.029	0.402	-0.003	-0.003	0.000	0.000	-0.012	0.211								
CN	0.364	0.035	0.035	0.050	0.040	0.382	-0.002	-0.002	-0.001	-0.001	-0.011	-0.121	0.311							
$COCH_3$ (I) LOC	0.073	-0.011	-0.007	0.010	0.070	-0.016	0.001	-0.001	0.000	0.001	0.000	-0.048	0.847	0.140	0.004	0.004	-0.005			
$COCH_3$ (I) MIN	0.264	-0.006	0.138	0.056	-0.070	0.233	0.000	-0.004	-0.001	0.002	-0.007	-0.055	0.432	0.020	-0.002	-0.002				
$CONH_2$	0.222	0.051	0.123	-0.004	-0.066	0.256	-0.001	-0.003	0.001	0.002	-0.008	-0.031	0.466	-0.007	-0.001	0.000				
COOH (II) Min	0.036	0.028	0.443	0.456	0.041	0.014	-0.001	-0.013	-0.014	-0.002	-0.001	-0.011	0.029	-0.005	0.000					
COOH (I) LOC	0.038	-0.001	-0.006	0.008	0.004	-0.010	0.000	-0.001	0.001	0.000	0.000	-0.051	0.946	0.074	-0.003					
F	0.325	0.089	0.090	-0.017	-0.017	0.461	-0.003	-0.003	0.000	0.000	-0.014	0.090								
H	0.459	0.029	0.029	0.029	0.029	0.459	-0.014	-0.001	-0.001	-0.001	-0.001	-0.014								
H (e)	-0.111	0.314	0.314	0.314	0.314	-0.111	-0.009	-0.009	-0.009	-0.009	0.002	0.002								
Li	0.985	-0.060	-0.060	0.059	0.059	-0.039	0.021	0.021	0.005	0.005	0.002	0.002								
$MeSO_2$	0.306	0.082	0.051	-0.034	-0.004	0.337	-0.002	0.000	-0.001	0.001	-0.010	-0.027	0.298	0.004	0.000	0.000	0.000	0.000		
Na	0.987	-0.061	-0.061	0.061	0.061	-0.041	0.021	0.021	0.005	0.005	0.002	0.001								
NC	0.330	0.073	0.073	-0.016	-0.016	0.411	-0.003	-0.003	0.000	0.000	-0.012	0.029	0.134							
NH2	0.087	0.206	0.206	-0.100	-0.100	0.403	-0.007	-0.007	0.003	0.003	-0.012	0.337	-0.009	-0.009						
NMe_2	-0.018	0.215	0.215	-0.107	-0.107	0.334	-0.007	-0.007	0.003	0.003	-0.010	0.448	-0.020	-0.020	0.004	0.025	0.011	0.004	0.025	0.011
NO_2 (I) LOC	-0.015	-0.011	-0.011	0.009	0.009	-0.029	0.000	0.000	0.000		0.001	-0.046	0.547	0.547						
NO_2 (I) MIN skew	0.366	0.032	0.030	0.013	0.015	0.412	-0.002	-0.002	-0.001	-0.001	-0.012	-0.025	0.087	0.089						
OH	0.217	0.133	0.176	-0.042	-0.083	0.438	-0.005	-0.006	0.001	0.002	-0.013	0.185	-0.005							
OMe	0.171	0.210	0.136	-0.105	-0.039	0.419	-0.007	-0.005	0.003	0.001	-0.013	0.223	-0.011	0.000	0.009	0.009				
Ph	0.110	0.131	0.131	-0.066	-0.066	0.272	-0.004	-0.004	0.002	0.002	-0.008	0.110	0.390							
SH	0.115	0.145	0.128	-0.075	-0.061	0.324	-0.005	-0.005	0.002	0.002	-0.010	0.450	-0.009							
SiH_3	0.433	0.014	0.090	0.046	-0.027	0.432	-0.001	-0.003	-0.002	0.000	-0.013	-0.024	0.000	0.029	0.026					
SMe	0.045	0.168	0.122	-0.092	-0.056	0.280	-0.006	-0.004	0.002	0.001	-0.009	0.534	-0.018	0.000	0.015	0.015				
tBu	0.367	0.052	0.147	0.020	-0.064	0.429	-0.002	-0.005	-0.001	0.001	-0.013	-0.002	0.071							

Table S4b. The NBO partial spins in the radical monocations of monosubstituted benzenes calculated using the U-DFT approximation, the ωB97XD functional and the aug-cc-pVTZ basis set. C(ipso), C(o), C(m), C(p), C(2o+2m), C(ip+p), Σ(C), Σ(H), X1 and R denote partial spin at C_{ipso} atom, averaged partial spin over C_{ortho} atoms, over C_{meta} atoms, sum of partial spin of all ortho and meta atoms, sum for the ipso and para atoms, sum of all carbon ring atoms, all ring H atoms, the atom attached in the ipso position and sum of partial spin of all substituent atoms, respectively.

subst	C(ipso)	C(o)	C(m)	C(p)	C(2o+2m)	C(ip+p)	Σ(C)	Σ(H)	X1	R
BF_2	-0.101	0.313	0.312	-0.107	1.248	-0.208	**1.040**	-0.036	-0.001	-0.004
BH_2	0.374	0.082	-0.014	0.437	0.135	0.811	**0.946**	-0.019	-0.010	0.074
$B(OH)_2$	0.458	0.039	0.015	0.436	0.107	0.894	**1.001**	-0.017	-0.017	0.019
Br	0.243	0.092	-0.036	0.367	0.112	0.610	**0.722**	-0.015	0.294	0.294
CCH	0.241	0.090	-0.040	0.322	0.100	0.563	**0.663**	-0.014	-0.057	0.351
CF_3(II)	0.033	0.241	0.239	0.031	0.958	0.064	**1.022**	-0.031	-0.002	0.005
CFO	0.029	-0.005	0.007	-0.014	0.004	0.015	**0.019**	0.001	-0.043	0.980
CH_3	0.378	0.085	-0.017	0.443	0.136	0.821	**0.957**	-0.019	0.004	0.062
CHO (I) LOC	0.073	-0.010	0.009	-0.021	-0.002	0.052	**0.050**	0.004	-0.044	0.947
CHO (I) MIN skew	0.324	0.066	0.067	0.280	0.265	0.604	**0.869**	-0.017	-0.077	0.147
Cl	0.285	0.089	-0.029	0.402	0.120	0.687	**0.807**	-0.018	0.211	0.211
CN	0.364	0.035	0.045	0.382	0.160	0.746	**0.906**	-0.017	-0.121	0.190
$COCH_3$ (I) LOC	0.073	-0.009	0.040	-0.016	0.062	0.057	**0.119**	0.001	-0.048	0.942
$COCH_3$ (I) MIN	0.264	0.066	-0.007	0.233	0.118	0.497	**0.615**	-0.010	-0.055	0.393
$CONH_2$	0.222	0.087	-0.035	0.256	0.104	0.478	**0.582**	-0.009	-0.031	0.427
COOH (II) Min	0.036	0.236	0.249	0.014	0.968	0.050	**1.018**	-0.031	-0.011	0.013
COOH (I) LOC	0.038	-0.004	0.006	-0.010	0.005	0.028	**0.033**	0.000	-0.051	0.966
F	0.325	0.090	-0.017	0.461	0.145	0.786	**0.931**	-0.020	0.090	0.090
H	0.459	0.029	0.029	0.459	0.116	0.918	**1.034**	-0.018	-0.014	-0.014
H (e)	-0.111	0.314	0.314	-0.111	1.256	-0.222	**1.034**	-0.034	0.002	0.002
Li	0.985	-0.060	0.059	-0.039	-0.002	0.946	**0.944**	0.054	0.002	0.002
$MeSO_2$	0.306	0.067	-0.019	0.337	0.095	0.643	**0.738**	-0.012	-0.027	0.275
Na	0.987	-0.061	0.061	-0.041	0.000	0.946	**0.946**	0.054	0.001	0.001
NC	0.330	0.073	-0.016	0.411	0.114	0.741	**0.855**	-0.018	0.029	0.163
NH_2	0.087	0.206	-0.100	0.403	0.212	0.490	**0.702**	-0.020	0.337	0.319
NMe_2	-0.018	0.215	-0.107	0.334	0.216	0.316	**0.532**	-0.018	0.448	0.488
NO_2 (I) LOC	-0.015	-0.011	0.009	-0.029	-0.004	-0.044	**-0.048**	0.001	-0.046	1.048
NO_2 (I) MIN skew	0.366	0.031	0.014	0.412	0.090	0.778	**0.868**	-0.018	-0.025	0.151
OH	0.217	0.155	-0.063	0.438	0.184	0.655	**0.839**	-0.021	0.185	0.180
OMe	0.171	0.173	-0.072	0.419	0.202	0.590	**0.792**	-0.021	0.223	0.230
Ph	0.110	0.131	-0.066	0.272	0.130	0.382	**0.512**	-0.012	0.110	0.500
SH	0.115	0.137	-0.068	0.324	0.137	0.439	**0.576**	-0.016	0.450	0.441

SiH_3	0.433	0.052	0.010	0.432	0.123	0.865	**0.988**	-0.019	-0.024	0.031
SMe	0.045	0.145	-0.074	0.280	0.142	0.325	**0.467**	-0.016	0.534	0.546
tBu	0.367	0.100	-0.022	0.429	0.155	0.796	**0.951**	-0.020	-0.002	0.069

Table S4c. The NBO partial spins in the radical monocations of monosubstituted benzenes calculated using the RO-DFT approximation, the ωB97XD functional and the aug-cc-pVTZ basis set.

subst	C1	C2	C6	C3	C5	C4	H2	H6	H3	H5	H4	X1	X2	X3	X3	X4	X5	X6	X7	X8
BF_2 (e) MIN	0.000	0.256	0.243	0.243	0.257	0.000	0.000	0.000	0.000	0.000	0.000	0.000	0.000	0.000						
BH_2	0.307	0.090	0.090	0.053	0.053	0.324	0.000	0.000	0.000	0.000	0.000	0.022	0.031	0.031						
$B(OH)_2$	0.372	0.075	0.074	0.062	0.062	0.324	0.000	0.000	0.000	0.000	0.000	0.003	0.013	0.013	0.000	0.000				
Br	0.234	0.084	0.084	0.038	0.038	0.240	0.000	0.000	0.000	0.000	0.000	0.283								
CCH	0.236	0.078	0.078	0.030	0.030	0.190	0.000	0.000	0.000	0.000	0.000	0.087	0.271	0.000						
CF_3 (II)	0.072	0.349	0.069	0.067	0.358	0.077	0.000	0.000	0.000	0.000	0.000	0.000	0.000	0.004	0.004					
CFO	0.023	0.005	0.001	0.003	0.000	0.000	0.000	0.000	0.001	0.000	0.000	0.009	0.918	0.040						
CH_3	0.310	0.092	0.092	0.052	0.052	0.330	0.000	0.000	0.000	0.000	0.000	0.027	0.007	0.032	0.007					
CHO	0.123	0.030	0.335	0.331	0.037	0.115	0.000	0.000	0.000	0.000	0.000	0.000	0.028	0.000						
Cl	0.256	0.087	0.087	0.044	0.044	0.279	0.000	0.000	0.000	0.000	0.000	0.000	0.203							
CN	0.310	0.066	0.066	0.053	0.053	0.269	0.000	0.000	0.000	0.000	0.000	0.000	0.023	0.159						
$COCH_3$	0.256	0.094	0.028	0.008	0.064	0.129	0.001	0.001	0.000	0.001	0.000	0.015	0.381	0.019	0.001	0.001	0.000			
$CONH_2$	0.240	0.082	0.049	0.015	0.041	0.138	0.001	0.001	0.000	0.001	0.000	0.010	0.414	0.007	0.001	0.000				
COOH (II) MIN	0.070	0.071	0.345	0.352	0.077	0.067	0.000	0.000	0.000	0.000	0.000	0.000	0.017	0.000	0.000					
COOH (I) LOC	0.007	0.256	0.203	0.189	0.304	0.008	0.000	0.000	0.000	0.000	0.000	0.000	0.000	0.003	0.000					
F	0.269	0.094	0.094	0.054	0.054	0.352	0.000	0.000	0.000	0.000	0.000	0.083								
H	0.357	0.071	0.071	0.071	0.071	0.357	0.000	0.000	0.000	0.000	0.000	0.000								
H (e) imag	0.000	0.250	0.250	0.250	0.250	0.000	0.000	0.000	0.000	0.000	0.000	0.000								
Li	0.876	0.010	0.010	0.028	0.028	0.001	0.016	0.016	0.004	0.004	0.001	0.004								
$MeSO_2$	0.305	0.052	0.079	0.047	0.041	0.213	0.001	0.002	-0.001	-0.002	0.006	0.013	0.009	0.227	0.005	0.000	0.000	0.000		
Na	0.876	0.011	0.011	0.029	0.029	0.001	0.016	0.016	0.004	0.004	0.001	0.003								
NC	0.282	0.083	0.083	0.048	0.048	0.293	0.000	0.000	0.000	0.000	0.000	0.072	0.089							
NH2	0.145	0.137	0.137	0.022	0.022	0.270	0.000	0.000	0.000	0.000	0.000	0.266	0.000	0.000						
NMe_2	0.095	0.129	0.129	0.014	0.014	0.187	0.000	0.000	0.000	0.000	0.000	0.363	0.006	0.002	0.008	0.018	0.006	0.002	0.008	0.018
NO_2 (I) MIN	0.002	0.248	0.248	0.240	0.240	0.000	0.000	0.000	0.000	0.000	0.000	0.000	0.011	0.011						
NO_2 (I) LOC	0.000	0.208	0.208	0.208	0.209	0.000	0.000	0.000	0.000	0.000	0.000	0.000	0.083	0.083						
OH	0.212	0.106	0.133	0.048	0.027	0.318	0.000	0.000	0.000	0.000	0.000	0.156								
OMe	0.190	0.149	0.105	0.017	0.050	0.296	0.000	0.000	0.000	0.000	0.000	0.180	0.002	0.006	0.006	0.000				
Ph	0.149	0.083	0.083	0.017	0.017	0.149	0.000	0.000	0.000	0.000	0.000	0.149	0.349							
SH	0.161	0.088	0.099	0.026	0.020	0.189	0.000	0.000	0.000	0.000	0.000	0.417	0.000							
SiH_3	0.349	0.059	0.100	0.083	0.042	0.323	0.000	0.000	0.000	0.000	0.000	0.007	0.018	0.020						
SMe	0.117	0.077	0.101	0.025	0.009	0.147	0.000	0.000	0.000	0.000	0.000	0.498	0.003	0.012	0.012	0.000				
*t*Bu	0.304	0.072	0.124	0.074	0.029	0.316	0.000	0.000	0.000	0.000	0.000	0.017	0.062							

Table S4d. The NBO partial spins in the radical monocations of monosubstituted benzenes calculated using the RO-DFT approximation, the ωB97XD functional and the aug-cc-pVTZ basis set. C(ipso), C(o), C(m), C(p), C(2o+2m), C(ip+p), Σ(C), Σ(H), X1 and R denote partial spin at C_{ipso} atom, averaged partial spin over C_{ortho} atoms, over C_{meta} atoms, sum of partial spin of all ortho and meta atoms, sum for the ipso and para atoms, sum of all carbon ring atoms, all ring H atoms, the atom attached in the ipso position and sum of partial spin of all substituent atoms, respectively.

subst	C(ipso)	C(o)	C(m)	C(p)	C(2o+2m)	C(ip+p)	Σ(C)	Σ(H)	X1	R
BF_2 (e) MIN	0.000	0.250	0.250	0.000	0.999	0.000	**0.999**	0.000	0.000	0.000
BH_2	0.307	0.090	0.053	0.324	0.286	0.631	**0.917**	0.000	0.022	0.084
$B(OH)_2$	0.372	0.075	0.062	0.324	0.273	0.696	**0.969**	0.000	0.003	0.029
Br	0.234	0.084	0.038	0.240	0.244	0.474	**0.718**	0.000	0.283	0.283
CCH	0.236	0.078	0.030	0.190	0.216	0.426	**0.642**	0.000	0.087	0.358
CF_3 (II)	0.072	0.209	0.213	0.077	0.843	0.149	**0.992**	0.000	0.000	0.008
CFO	0.023	0.003	0.002	0.000	0.009	0.023	**0.032**	0.001	0.009	0.967
CH_3	0.310	0.092	0.052	0.330	0.288	0.640	**0.928**	0.000	0.027	0.073
CHO	0.123	0.183	0.184	0.115	0.733	0.238	**0.971**	0.000	0.000	0.028
Cl	0.256	0.087	0.044	0.279	0.262	0.535	**0.797**	0.000	0.000	0.203
CN	0.310	0.066	0.053	0.269	0.238	0.579	**0.817**	0.000	0.000	0.182
$COCH_3$	0.256	0.061	0.036	0.129	0.194	0.385	**0.579**	0.003	0.015	0.417
$CONH_2$	0.240	0.066	0.028	0.138	0.187	0.378	**0.565**	0.003	0.010	0.432
COOH (II) MIN	0.070	0.208	0.215	0.067	0.845	0.137	**0.982**	0.000	0.000	0.017
COOH (I) LOC	0.007	0.230	0.247	0.008	0.952	0.015	**0.967**	0.000	0.000	0.003
F	0.269	0.094	0.054	0.352	0.296	0.621	**0.917**	0.000	0.083	0.083
H	0.357	0.071	0.071	0.357	0.284	0.714	**0.998**	0.000	0.000	0.000
H (e) imag	0.000	0.250	0.250	0.000	1.000	0.000	**1.000**	0.000	0.000	0.000
Li	0.876	0.010	0.028	0.001	0.076	0.877	**0.953**	0.041	0.004	0.004
$MeSO_2$	0.305	0.066	0.044	0.213	0.219	0.518	**0.737**	0.006	0.013	0.254
Na	0.876	0.011	0.029	0.001	0.080	0.877	**0.957**	0.041	0.003	0.003
NC	0.282	0.083	0.048	0.293	0.262	0.575	**0.837**	0.000	0.072	0.161
NH2	0.145	0.137	0.022	0.270	0.318	0.415	**0.733**	0.000	0.266	0.266
NMe_2	0.095	0.129	0.014	0.187	0.286	0.282	**0.568**	0.000	0.363	0.431
NO_2 (I) MIN	0.002	0.248	0.240	0.000	0.976	0.002	**0.978**	0.000	0.000	0.022
NO_2 (I) LOC	0.000	0.208	0.209	0.000	0.833	0.000	**0.833**	0.000	0.000	0.166
OH	0.212	0.120	0.038	0.318	0.314	0.530	**0.844**	0.000	0.156	0.156
OMe	0.190	0.127	0.034	0.296	0.321	0.486	**0.807**	0.000	0.180	0.194
Ph	0.149	0.083	0.017	0.149	0.200	0.298	**0.498**	0.000	0.149	0.498
SH	0.161	0.094	0.023	0.189	0.233	0.350	**0.583**	0.000	0.417	0.417
SiH_3	0.349	0.080	0.063	0.323	0.284	0.672	**0.956**	0.000	0.007	0.045
SMe	0.117	0.089	0.017	0.147	0.212	0.264	**0.476**	0.000	0.498	0.525
*t*Bu	0.304	0.098	0.052	0.316	0.299	0.620	**0.919**	0.000	0.017	0.079

Substituent	(α + β) valenece orbitals populations				α valenece orbitals populations				β valenece orbitals populations				spin popul	
	(α + β)(*s*+*p*x+*pz*)	(α + β)(*py*)	sEDA(*D*)	pEDA(*D*)	α (*s*+*p*x+*pz*)	α (*py*)	sEDA(*D* α)	pEDA(*D* α)	β (*s*+*p*x+*pz*)	β (*py*)	sEDA(*D* β)	pEDA(*D* β)	σ	π
BF_2	19.544	4.960	0.142	-0.030	9.790	2.982	0.071	-0.013	9.755	1.978	0.071	-0.018	0.035	1.004
BH_2	19.491	5.105	0.089	0.115	9.761	3.010	0.042	0.015	9.730	2.096	0.046	0.100	0.031	0.914
$B(OH)_2$	19.493	4.995	0.091	0.005	9.763	2.981	0.044	-0.014	9.730	2.013	0.046	0.017	0.033	0.968
Br	19.218	5.347	-0.184	0.357	9.620	3.025	-0.099	0.030	9.598	2.322	-0.086	0.326	0.022	0.703
CCH	19.153	5.308	-0.249	0.318	9.587	2.975	-0.132	-0.020	9.566	2.333	-0.118	0.337	0.021	0.642
CF_3	19.266	4.996	-0.136	0.006	9.650	2.993	-0.069	-0.002	9.616	2.002	-0.068	0.006	0.034	0.991
CFO	19.091	5.680	-0.311	0.690	9.564	2.831	-0.155	-0.164	9.527	2.850	-0.157	0.854	0.037	-0.019
CH_3	19.168	5.092	-0.234	0.102	9.600	3.008	-0.119	0.013	9.568	2.084	-0.116	0.088	0.032	0.924
CHO (I) LOC	19.056	5.679	-0.346	0.689	9.566	2.826	-0.153	-0.169	9.490	2.853	-0.194	0.857	0.076	-0.027
CHO (I) MIN skew	19.247	5.114	-0.155	0.124	9.647	2.968	-0.072	-0.027	9.600	2.146	-0.084	0.150	0.047	0.822
Cl	19.153	5.278	-0.249	0.288	9.589	3.032	-0.130	0.037	9.564	2.247	-0.120	0.251	0.025	0.785
CN	19.201	5.146	-0.201	0.156	9.614	2.972	-0.105	-0.023	9.587	2.174	-0.097	0.178	0.027	0.798
$COCH_3$ (I) LOC	19.031	5.746	-0.371	0.756	9.552	2.864	-0.167	-0.131	9.479	2.882	-0.205	0.886	0.073	-0.018
$COCH_3$ (I) MIN skew	19.114	5.428	-0.288	0.438	9.602	2.977	-0.117	-0.018	9.512	2.451	-0.172	0.455	0.090	0.526
$CONH_2$	19.082	5.483	-0.320	0.493	9.580	2.993	-0.139	-0.002	9.502	2.490	-0.182	0.494	0.078	0.503
COOH (II) MIN	19.293	4.986	-0.109	-0.004	9.664	2.984	-0.055	-0.011	9.629	2.002	-0.055	0.006	0.035	0.982
COOH (I) LOC	19.046	5.749	-0.356	0.759	9.545	2.869	-0.174	-0.126	9.501	2.880	-0.183	0.884	0.044	-0.011
F	18.808	5.159	-0.594	0.169	9.416	3.033	-0.303	0.038	9.392	2.126	-0.292	0.130	0.024	0.907
H	19.402	4.990	0.000	0.000	9.719	2.995	0.000	0.000	9.684	1.996	0.000	0.000	0.035	0.999
H (e) imag	19.403	4.990	0.001	0.000	9.719	2.995	0.000	0.000	9.684	1.996	0.000	0.000	0.035	0.999
Li	19.162	5.908	-0.240	0.918	10.048	2.955	0.329	-0.040	9.114	2.953	-0.570	0.957	0.934	0.002
$MESO_2$	19.295	5.325	-0.107	0.335	9.681	2.998	-0.038	0.003	9.614	2.327	-0.070	0.331	0.067	0.671
Na	19.124	5.956	-0.278	0.966	10.029	2.979	0.310	-0.016	9.095	2.977	-0.589	0.981	0.934	0.002
NC	18.971	5.172	-0.431	0.182	9.497	3.001	-0.222	0.006	9.473	2.171	-0.211	0.175	0.024	0.830
NH_2	18.911	5.467	-0.491	0.477	9.463	3.077	-0.256	0.082	9.448	2.390	-0.236	0.394	0.015	0.687
NMe_2	18.861	5.618	-0.541	0.628	9.434	3.071	-0.285	0.076	9.427	2.548	-0.257	0.552	0.007	0.523
NO_2 (I) MIN	19.033	5.146	-0.369	0.156	9.531	2.992	-0.188	-0.003	9.502	2.154	-0.182	0.158	0.029	0.838

NO_2 (I) LOC	18.906	5.623	-0.496	0.633	9.453	2.787	-0.266	-0.208	9.453	2.836	-0.231	0.840	0.000	-0.049
OH	18.849	5.294	-0.553	0.304	9.434	3.056	-0.285	0.061	9.414	2.238	-0.270	0.242	0.020	0.818
OMe	18.830	5.349	-0.572	0.359	9.424	3.060	-0.295	0.065	9.406	2.288	-0.278	0.292	0.018	0.772
Ph	19.070	5.489	-0.332	0.499	9.543	2.993	-0.176	-0.002	9.527	2.496	-0.157	0.500	0.016	0.497
SH	19.215	5.514	-0.187	0.524	9.614	3.039	-0.105	0.044	9.601	2.475	-0.083	0.479	0.013	0.564
SiH_3	[illegible]	[illegible]	[illegible]	[illegible]	[illegible]	[illegible]	[illegible]	[illegible]	[illegible]	[illegible]	[illegible]	[illegible]	[illegible]	[illegible]
SMe	[illegible]	[illegible]	[illegible]	[illegible]	[illegible]	[illegible]	[illegible]	[illegible]	[illegible]	[illegible]	[illegible]	[illegible]	[illegible]	[illegible]
*t*Bu	19.159	5.094	-0.243	0.104	9.595	3.005	-0.124	0.010	9.564	2.088	-0.120	0.092	0.031	0.917

Substituent	(α + β) valenece orbitals populations				α valenece orbitals populations				β valenece orbitals populations				spin popul.	
	(α + β)(s+p_x+p_z)	(α + β)(p_y)	sEDA(D)	pEDA(D)	α (s+p_x+p_z)	α (p_y)	sEDA(D α)	pEDA(D α)	β (s+p_x+p_z)	β (p_y)	sEDA(D β)	pEDA(D β)	σ	π
BF_2	19.547	4.961	0.142	-0.029	9.773	2.980	0.071	-0.015	9.773	1.981	0.071	-0.015	0.000	0.999
BH_2	19.492	5.108	0.087	0.118	9.746	3.012	0.044	0.017	9.746	2.097	0.044	0.101	0.000	0.915

Table S5a. The NBO valence ring orbital electron and spin populations in the radical monocations of monosubstituted benzenes calculated using the U-DFT approximation, the ωB97XD functional and the aug-cc-pVTZ basis set.

Table S5b. The NBO valence ring orbital electron and spin populations in the radical monocations of monosubstituted benzenes calculated using the RO-DFT approximation, the ωB97XD functional and the aug-cc-pVTZ basis set. The *y* axis is perpendicular to the phenyl plane.

$B(OH)_2$	19.495	4.996	0.090	0.006	9.748	2.982	0.046	-0.013	9.748	2.013	0.046	0.017	0.000	0.969
Br	19.220	5.348	-0.185	0.358	9.610	3.032	-0.092	0.037	9.610	2.317	-0.092	0.321	0.000	0.715
CCH	19.153	5.310	-0.252	0.320	9.577	2.975	-0.125	-0.020	9.577	2.335	-0.125	0.339	0.000	0.640
CF_3	19.268	4.996	-0.137	0.006	9.634	2.993	-0.068	-0.002	9.634	2.003	-0.068	0.007	0.000	0.990
CFO	19.091	5.680	-0.314	0.690	9.561	2.840	-0.141	-0.155	9.530	2.840	-0.172	0.844	0.031	0.000
CH_3	19.170	5.095	-0.235	0.105	9.585	3.010	-0.117	0.015	9.585	2.084	-0.117	0.088	0.000	0.926
CHO	19.301	4.988	-0.104	-0.002	9.650	2.979	-0.052	-0.016	9.650	2.009	-0.052	0.013	0.000	0.970
Cl	19.154	5.280	-0.251	0.290	9.577	3.038	-0.125	0.043	9.577	2.242	-0.125	0.246	0.000	0.796
CN	19.203	5.138	-0.202	0.148	9.602	2.977	-0.100	-0.018	9.602	2.161	-0.100	0.165	0.000	0.816
$COCH_3$	19.102	5.454	-0.303	0.464	9.591	2.976	-0.111	-0.019	9.511	2.479	-0.191	0.483	0.080	0.497
$CONH_2$	19.079	5.490	-0.326	0.500	9.571	2.994	-0.131	-0.001	9.508	2.496	-0.194	0.500	0.063	0.498
COOH (II) MIN	19.295	4.987	-0.110	-0.003	9.648	2.984	-0.054	-0.011	9.648	2.003	-0.054	0.007	0.000	0.981
COOH (I) LOC	19.274	4.961	-0.131	-0.029	9.637	2.978	-0.065	-0.017	9.637	1.983	-0.065	-0.013	0.000	0.995
F	18.810	5.159	-0.595	0.169	9.405	3.037	-0.297	0.042	9.405	2.122	-0.297	0.126	0.000	0.915
H	19.405	4.990	0.000	0.000	9.702	2.995	0.000	0.000	9.702	1.996	0.000	0.000	0.000	0.999
H (e)	19.410	4.990	0.005	0.000	9.703	2.994	0.001	-0.001	9.703	1.996	0.001	0.000	0.000	0.998
Li	19.162	5.908	-0.243	0.918	10.054	2.954	0.352	-0.041	9.108	2.954	-0.594	0.958	0.946	0.000
$MESO_2$	19.302	5.311	-0.103	0.321	9.672	3.001	-0.030	0.006	9.630	2.310	-0.072	0.314	0.042	0.691
Na	19.124	5.956	-0.281	0.966	10.035	2.978	0.333	-0.017	9.089	2.978	-0.613	0.982	0.946	0.000
NC	18.972	5.175	-0.433	0.185	9.486	3.006	-0.216	0.011	9.486	2.169	-0.216	0.173	0.000	0.837
NH_2	18.916	5.455	-0.489	0.465	9.458	3.092	-0.244	0.097	9.458	2.362	-0.244	0.366	0.000	0.730
NMe_2	18.866	5.609	-0.539	0.619	9.433	3.085	-0.269	0.090	9.432	2.523	-0.270	0.527	0.001	0.562
NO_2 (I) MIN	19.060	5.033	-0.345	0.043	9.529	3.003	-0.173	0.008	9.527	2.030	-0.175	0.034	0.002	0.973
NO_2 (I) LOC	19.048	5.087	-0.357	0.097	9.524	2.960	-0.178	-0.035	9.524	2.960	-0.178	0.964	0.000	0.000
OH	18.851	5.290	-0.554	0.300	9.426	3.066	-0.276	0.071	9.426	2.224	-0.276	0.228	0.000	0.842
OMe	18.833	5.341	-0.572	0.351	9.417	3.072	-0.285	0.077	9.417	2.269	-0.285	0.273	0.000	0.803
Ph	19.071	5.490	-0.334	0.500	9.536	2.993	-0.166	-0.002	9.535	2.497	-0.167	0.501	0.001	0.496
SH	19.217	5.510	-0.188	0.520	9.609	3.046	-0.093	0.051	9.609	2.464	-0.093	0.468	0.000	0.582
SiH_3	19.604	5.039	0.199	0.049	9.802	2.996	0.100	0.001	9.802	2.043	0.100	0.047	0.000	0.953
SMe	19.198	5.615	-0.207	0.625	9.599	3.044	-0.103	0.049	9.599	2.571	-0.103	0.575	0.000	0.473
*t*Bu	19.160	5.098	-0.245	0.108	9.580	3.007	-0.122	0.012	9.580	2.091	-0.122	0.095	0.000	0.916

Table S6. The σ˙ values for the substituents considered in this study according to Hansch and Gao.[61]

Substituent	Dst&Arnold		Jiang&Ji		Creary et al.		Comment
	para	meta	para	meta	para	meta	
BF_2							
BH_2							
$B(OH)_2$					*0.280*		*-$B(OCH_2)_2$*
Br			0.230	0.120	0.130		
CCH							
CF_3	-0.090	-0.170	-0.010	-0.070	0.080	-0.070	
CFO							
CH_3	0.150	0.020	0.150	0.000	0.110	0.030	
CHO							
Cl	0.110	-0.070	0.220	-0.050	0.120	-0.040	
CN	0.400	-0.260	0.420	0.110	0.460	-0.120	
$COCH_3$	0.600		0.540				
$CONH_2$			0.380				
COOH			0.380				
F	-0.110	-0.090	-0.020	0.030	-0.080	-0.050	
H	0.000	0.000	0.000	0.000	0.000	0.000	
Li							
$MeSO_2$	0.050		0.380		0.180	-0.070	
Na							
NC							
NH_2							
NMe_2			1.000		0.900		
NO_2			0.360	0.000	0.570	-0.110	
OH							
OMe	0.180	-0.010	0.230	0.100	0.240	-0.020	
Ph			0.470		0.460		
SH							
SiH_3	*0.170*		*0.310*	*0.000*	*0.180*	*0.030*	*$SiMe_3$*
SMe	0.630		0.620		0.430	-0.030	
tBu	0.080		0.260	0.110	0.130		
